# Pulmonary hazards of nanoplastic particles: a study using polystyrene in in vitro models of the alveolar and bronchial epithelium

**DOI:** 10.1186/s12951-025-03419-6

**Published:** 2025-05-28

**Authors:** Sara Michelini, Safaa Mawas, Ema Kurešepi, Francesco Barbero, Katarina Šimunović, Dorian Miremont, Stéphanie Devineau, Martin Schicht, Victor Ganin, Øyvind Pernell Haugen, Anani Komlavi Afanou, Charlotte Izabelle, Shan Zienolddiny-Narui, Katharina Jüngert, Neža Repar, Ivana Fenoglio, Barbara Šetina Batić, Friedrich Paulsen, Ines Mandić-Mulec, Sonja Boland, Andreja Erman, Damjana Drobne

**Affiliations:** 1 Biotechnical Faculty, Department of Biology, University of Ljubljana, Ljubljana, Slovenia; 2 Université Paris Cité, CNRS, Unité de Biologie Fonctionnelle et Adaptative, Paris, France; 3 Department of Chemistry, Laboratory of Toxicity and Biocompatibility of Materials, University of Torino, Torino, Italy; 4 Biotechnical Faculty, Department of Microbiology, University of Ljubljana, Ljubljana, Slovenia; 5 Institute of Functional and Clinical Anatomy, Friedrich-Alexander-University of Erlangen-Nürnberg, Erlangen, Germany; 6 Institute of Metals and Technology, Ljubljana, Slovenia; 7 STAMI, National Institute of Occupational Health, Oslo, Norway; 8 Université Paris Cité, CNRS UAR612, Inserm US25, Cellular and Molecular Imaging Facility, Paris, France; 9 Faculty of Medicine, Institute of Cell Biology, University of Ljubljana, Ljubljana, Slovenia

**Keywords:** Nanoplastic, Surfactant protein, Mucin, Lamellar body, A549, Calu-3, Polystyrene, Alveoli, Bronchi, Transwells

## Abstract

**Background:**

Nanoplastics (NPs) are released into the environment through the degradation of plastic objects, leading to human exposure. Due to their small size, concerns have been raised about the potential hazards to the respiratory tract, as ultrafine and nanoparticles are known to penetrate till the alveolar regions of the lungs, potentially impairing their functions. Thus, in the present study, we used model polystyrene nanoparticles doped with the fluorescent metal europium (PS-Eu) to enhance the understanding of NPs hazard and investigate adverse outcomes associated with exposure in human lungs using alveolar (A549) and bronchial (Calu-3) cell models grown in 2D and 3D submerged conditions or quasi air-liquid interface (ALI) conditions (3D).

**Results:**

Briefly, after in-dept physicochemical characterization of the particles, we assessed their impact on ROS production, cell viability (AlamarBlue and lactate dehydrogenase assays) and barrier integrity (lucifer yellow assay and TEER measurement), finding no negative effects in either model. However, in alveolar cells, particles increased acidic organelle activity. Transmission electron microscopy and Raman microscopy showed, in both models, a dose- and cell-dependent particle uptake with PS-Eu accumulating in numerous and large endo-lysosomes, which, in transwells-grown A549 cells, often contained also lamellar bodies (LBs), organelles involved in surfactants storage and secretion. After extensively quantifying surfactant proteins (SP) in the pellet and supernatant fractions of treated A549 cells, we observed a significant reduction in several members of this family, including surfactant protein B, which is crucial for lamellar body formation and surface tension regulation in the lungs. In quasi-ALI Calu-3 cultures instead, PS-Eu significantly upregulated interleukin 6 (IL-6) and increased transforming growth factor beta β (TGF-β), zonula occludens 1 (ZO-1), and mucin (MUC) 5B mRNA expressions causing a moderate proinflammatory response.

**Conclusion:**

Our results show that PS-Eu exposure does not induce acute cytotoxicity in these models, but affects cell-specific functions like surfactant, mucin, and cytokine production. This underscores the limitations of relying solely on standard cytotoxicity tests for particle hazard assessment and highlights the importance of investigating cell function-specific signaling pathways. To support researchers in hazard assessment, we propose specific classes of biomarkers to test in in vitro lung models.

**Supplementary Information:**

The online version contains supplementary material available at 10.1186/s12951-025-03419-6.

## Graphical abstract

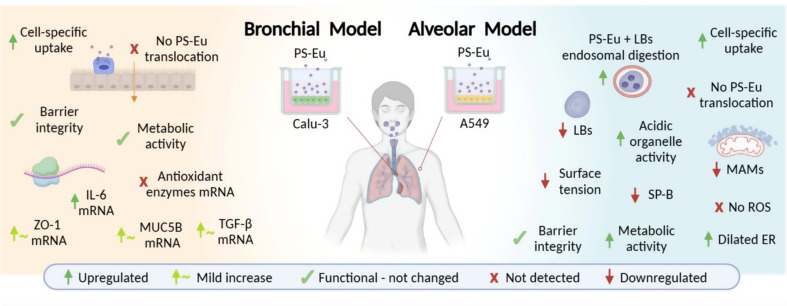

## Background

Plastic production is constantly increasing worldwide, resulting in a large amount of plastic waste, only some of which is successfully collected for recycling, landfilling or incineration [1]. As a result, large quantities of plastics are released into the environment, where abiotic (e.g. abrasion of tire wear) and biotic degradation slowly breaks them down into small fragments, known as micro- and nanoplastics (MPs/NPs) [1–4]. Alternatively, MPs/NPs can be released from products that contain these particles as part of their formulation, such as grinding material and personal care products [4–8]. Microplastics are defined as particles < 5 mm in size and once the particles are smaller than 1 μm, they are referred to as nanoplastics. For both size classes of particles, uptake by organisms and trophic transfer have already been demonstrated in various ecosystems. The smaller the particles become, the higher the probability that they will transfer into the tissue causing harmful effects [1–4, 9]. In this context, Triebskorn et al. conducted a comprehensive review of existing toxicity data on MPs/NPs and concluded that the risk posed by larger MPs is relatively low when compared to smaller MPs, just a few micrometres in diameter, and NPs [10].

The small size and widespread distribution of MPs/NPs have raised concerns about the potential hazards to the respiratory tract, as particles smaller than 10 μm can enter the airways and those smaller than 1–3 μm can reach the alveoli, potentially impairing the gas exchange properties of this organ [11, 12]. Despite these concerns, the scientific understanding of the impacts of these small particles remains limited and incomplete.

A functional gas exchange surface is maintained through the secretion of surfactants by type II alveolar cells (ATII) and the maintenance of a continuous epithelial barrier. ATII cells constantly produce, store, secrete and recycle surfactants to regulate surface tension in the lungs and respond rapidly to environmental and physiological changes. Organelles that have important roles in this process are the lamellar bodies (LBs) that store surfactants before secretion, and the Golgi apparatus and the endoplasmic reticulum (ER), which both are involved in the production, distribution, and secretion of surfactants. Surfactants are a mixture of proteins and lipids whose main function is to reduce the surface tension of the water film at the interface between alveolar cells and the air. This is crucial to prevent atelectasis and allow lung expansion during inhalation, ultimately favouring gas exchange through the type I alveolar cells (ATI) [13, 14].

While most deposited nanosized plastic particles are found predominantly in the alveoli, a portion of them will be retained in the tracheobronchial region together with larger particles and their agglomerates [15]. Here they meet the bronchial mucosa, a multilayered tissue composed in the luminal part, by many ciliated and mucus-producing cells, which are involved in the capture and elimination of exogenous material through the process known as mucociliary clearance. In this process, deposited particles, pathogens, and other foreign substances are trapped within the mucus layer covering the epithelium. The coordinated beating of cilia then propels the mucus toward the digestive tract for removal [16, 17]. Unlike bronchi, the alveoli lack this defence mechanism; making NPs accumulate more likely in the lower airways, especially when the number of inhaled particles surpasses the clearance capacity of macrophages [17]. The bronchial epithelium has also immunomodulatory functions. By expressing cytokines, growth factors and mediators involved in cell-to-cell communication, it plays a pivotal role in regulating inflammatory processes.

Mucociliary clearance, surfactant production, and lung barrier dysfunctions together with unbalanced mucosal immune responses are linked to increased risk of infection and serve as markers of various diseases such cystic fibrosis, chronic obstructive pulmonary disease, primary ciliary dyskinesia, and others, highlighting their critical role in a healthy lung function [16–18]. In this context micro- and nanoparticles of various origins have been shown to cause lung damage in vitro, in vivo, and in exposed humans [19–22].

This study addresses the critical gap in understanding the effects and hazards of nanoplastics on human lung epithelial barriers, specifically the alveolar and bronchial epithelial barriers. To address this challenge, we selected polystyrene (PS), one of most produced polymer types [4, 6, 7, 23–25]^,^, which had been already detected in the lungs [26], as a model nanoplastic and conducted a study on its effects on two well-established cell models of the alveolar (adenocarcinoma human alveolar basal epithelial cells, A549) and bronchial epithelial barriers (cultured human airway epithelial cells, Calu-3) grown in different in vitro exposure systems characterized by different levels of complexities, such as the quasi air-liquid interface (ALI) which better resemble real-life conditions. To allow particle detection with Raman microscopy and spectroscopic methods we used NPs doped with europium, a fluorescent metal, referring to these particles as “PS-Eu”. These particles, which have a carboxylated surface, were thoroughly characterized both upon initial reception and under relevant culture conditions. This is important because properties such as surface charge, size, shape, and aggregation status can vary with environmental conditions, potentially altering their bio-identity and, as a result, their effects on cells. To assess the response of alveolar and bronchial models to PS-Eu, we investigated multiple biomarkers using biochemical methods, light and electron microscopy, spectroscopy, RT-qPCR, ELLA, and ELISA with the aim of improving the understanding of PS NPs hazard and mechanistic effects. Additionally, we propose different classes of biomarkers to assess nanoplastics hazard when using in vitro lung models.

## Results

### Nanoplastic characteristics and behaviour in the cell culture medium

**Fig. 1:**
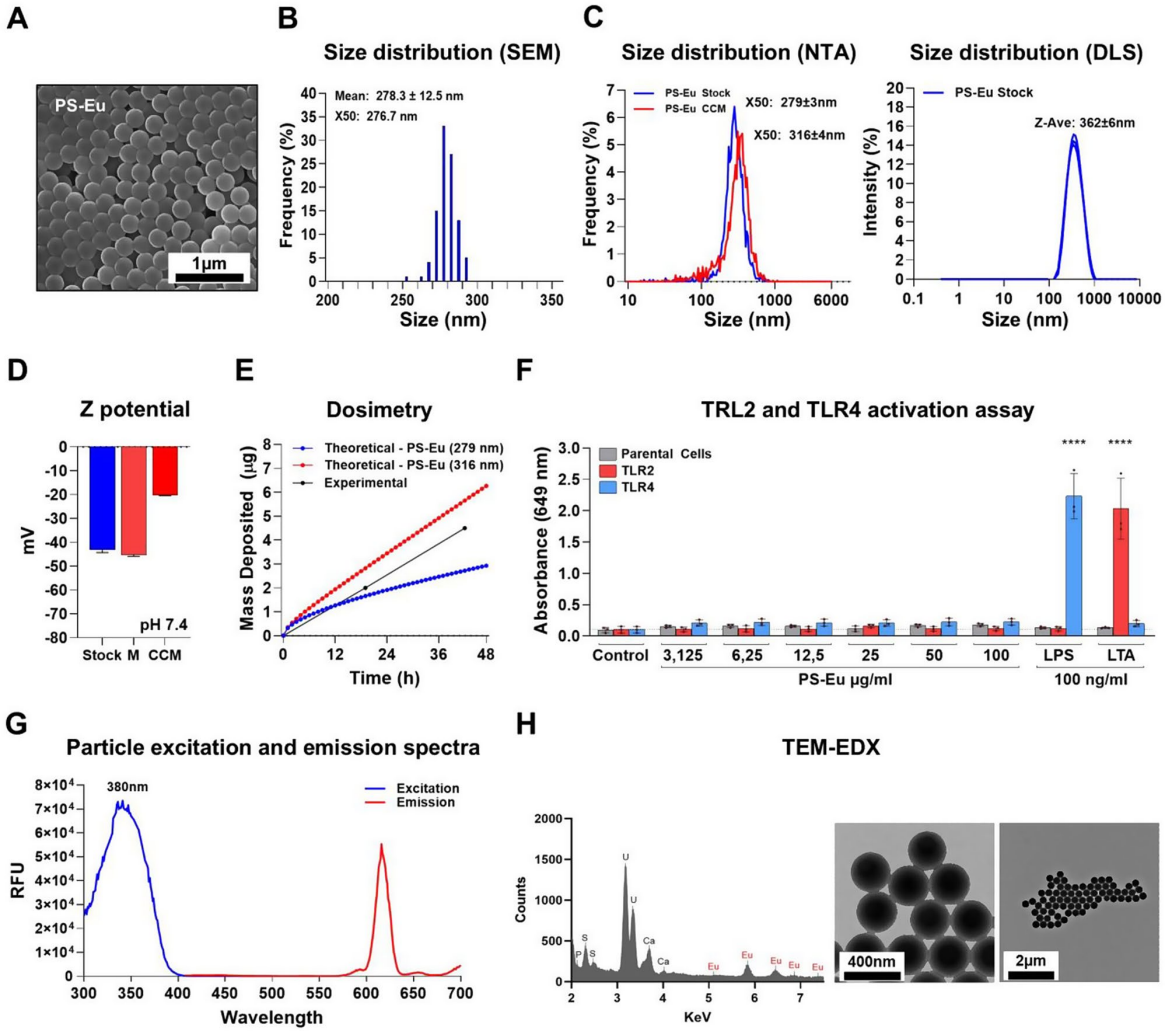
Particle characterization. **(A)** a representative SEM image of PS-Eu stock suspension and correspondent size distribution calculated using ImageJ on the same images **(B)**; **(C)** Left: size distribution of stock suspension (blue) or PS-Eu incubated for 48 h in CCM (red) calculated via NTA. Right: PS-Eu stock suspension size distribution calculated via DLS. (**D)** Z potential of PS-Eu stock suspension or PS-Eu incubated for 48 h in FBS-free medium (M) or in CCM. **(E)** PS-Eu deposited mass over 48 h calculated theoretically and experimentally combining the ISDD model and NTA. (**F)** Graph showing the level of TLR2 and TLR4 ligand contamination of PS-Eu stock suspension measured using the HEK293 reporter cell assay. 100 ng/ml of LPS and LTA were used as positive controls for TLR2 and TLR4 activation, respectively. Grey: parental cells. Red/Blue: cells expressing the human TLR2 or the TLR4 gene. **(G)** PS-Eu excitation and emission spectra in CCM. (**H)** TEM-EDX spectrum of PS-Eu stock showing the presence of the Eu element. Abbreviations: PS-Eu: europium doped polystyrene nanoparticles; SEM; Scanning electron microscopy; TEM: Transmission electron microscopy; CCM: Complete culture medium; NTA: nanoparticle tracking analysis; DLS: dynamic light scattering; FBS: fetal bovine serum; ISDD: in vitro sedimentation, diffusion and dosimetry; HEK293: Human embryonic kidney 293 cells; LPS: lipopolysaccharide; LTA: lipoteichoic acid; TLR: Toll-like receptor; RFU: relative fluorescence units; TEM-EDX: Transmission electron microscopy–energy dispersive X-ray

Since cell-particle interaction dynamics are strongly influenced by particle properties we characterized the particle both in stock suspension and in relevant culture conditions [27–29]. Figure 1.A, shows a scanning electron microscopy (SEM) image of the particle stock suspension which has a mean diameter of 278.3 ± 12.5 nm (calculated with ImageJ) (Fig. 1.B), and a negative Z potential of -43.1 ± 1.2 mV, as result of their carboxylated surface (Fig. 1.D). The SEM image shows monodisperse spherical particles and the size distribution analysis of the particle dispersion performed by nanoparticle tracking analysis (NTA) shows that PS-Eu hydrodynamic diameter (HD) presents a median value (X50) of 279 ± 3 nm and a mean size of 293 ± 2 nm (Fig. 1.C right). The size distribution evaluated by SEM was close enough to that measured by NTA to demonstrate that the particles in the dispersion were mainly single particles rather than aggregates. We also measured the HD via dynamic light scattering (DLS) and found a mean size (by intensity) of 362 ± 6 nm. The discrepancies found in this analysis are determined by the data collecting method, in fact, DLS overestimates large particles due to their higher intensity of the scattered light, whereas the NTA analysis does not have this limitation.

Since particle stock suspension contains the preservative sodium azide (0,05%), we washed the particles prior to exposure to Calu-3 cells. Subsequently, we measured the HD of the washed particles by DLS in water and found no significant changes when compared to the stock suspension (not shown). This suggests that particle washing procedure did not affect particle aggregation state.

However, particle size, charge, and aggregation state can change once they are exposed to the culture medium and consequently affect their behaviour and bio-identity [30]. To investigate these aspects, PS-Eu particles were incubated for 48 h either in A549 complete culture medium (CCM) or foetal bovine serum (FBS)-free culture medium (M), and then analysed by NTA, and electrophoresis light scattering (Fig. 1.C-E). In both cases, antibiotic-free mediums were used for submerged cultures as we previously demonstrated that the antibiotics present in culture media formulations can change anionic nanoparticle stability and uptake [30]. The results showed that after 48 h in CCM a protein corona was formed on the particle surface, causing an increase of the Z potential to -20.2 ± 0.3 mV (Fig. 1.D) without affecting their stability as shown by the size distribution analysis performed by NTA (Fig. 1.C). Here, in fact, we assist to an increase of the hydrodynamic diameter (~ 37 nm) without a deformation of the curve profile, which suggests that a protein corona is formed, and no clear aggregation occurred. In contrast, when particles were added to the FBS-free culture medium (M), they strongly aggregated and sedimented, a phenomenon that was clearly seen by DLS (Additional File 1). These particles maintained a strongly negative Z potential of -45.3 ± 0.6 mV even after being washed from the medium because their anionic surface was not covered by culture medium proteins as occurred in the CCM sample (Fig. 1.D). Together, these data suggest that the presence of FBS in A549 medium lead to the formation of a nanoparticle protein corona which is critical for particle colloidal stability under submerged culture conditions.

Particle aggregation and interaction with proteins can strongly alter their dosimetry by changing their HD and their overall density, making them more or less susceptible to gravitational settling and Brownian force. To estimate the extent of particle deposition in our submerged A549 models, we conducted both a theoretical and experimental dosimetric analysis over 48 h in CCM. In the theoretical calculations, we considered PS-Eu either with or without a hard protein corona on their surface, as determined by NTA [31]. The deposited dose was estimated using the in vitro sedimentation, diffusion and dosimetry (ISDD) model in both analysis and the data are shown in Fig. 1.E [32]. Our theoretical analysis showed, as expected, that particles with a hard corona deposited quicker in comparison to PS-Eu without protein corona, meaning that the same applied dose of particles leads to higher deposited dose when particles are in CCM. Notably, the experimentally measured deposited fraction falls between the theoretically calculated deposited fraction for pristine PS-Eu and PS-Eu with protein corona (Fig. 1.E.), suggesting that the formed protein corona is not as compact as theoretically assumed for PS-Eu in CCM. Our results revealed a quite linear correlation between time and deposited mass, with ~ 20% deposited at 48 h. A more precise description of the process can be found in the material and methods section.

To exclude bacteria-derived ligand contamination of the particles during synthesis, we screened the PS-Eu particles for Toll-like receptor 2 (TLR2) and 4 (TLR4) ligands using human embryonic kidney 293 (HEK293) reporter cells and found the particles free from these stimulatory molecules (Fig. 1.F). This is critical as many misleading results regarding particle hazard were obtained from assessing the effects of contaminated metallic particles [33]. Human cells are in fact, susceptible to these compounds and can react to them through the TLR2 and TLR4 signalling pathways, among others. Between the cellular effects that can be induced by LPS we can find ROS production, apoptosis and loss of mitochondria membrane potential, cytokines production and cell migration [34, 35], which are effects commonly detected during particle treatment [36]. Thus, in this context, the use of this assay is particularly relevant as it more closely reflects the natural response of cells to endotoxins when compared, for example, to a standard limulus amoebocyte lysate assay (LAL) [37, 38].

Finally, since the particles used in this study are loaded with europium, which is a fluorescent heavy metal that can allow particle detection by various methods, we recorded the absorbance and emission spectra of PS-Eu particles in CCM (Fig. 1.G) and found that the maximum emission was at 613 nm. Transmission electron microscopy–energy dispersive X-ray analysis (TEM-EDX) was used to confirm Eu presence in our particles, with a europium’s main electronic transition at 5.85 KeV (Fig. 1.H).

### Impact of PS-Eu on human alveolar models

#### PS-Eu affect acidic organelle activity in A549 cells grown under standard 2D submerged conditions

**Fig. 2:**
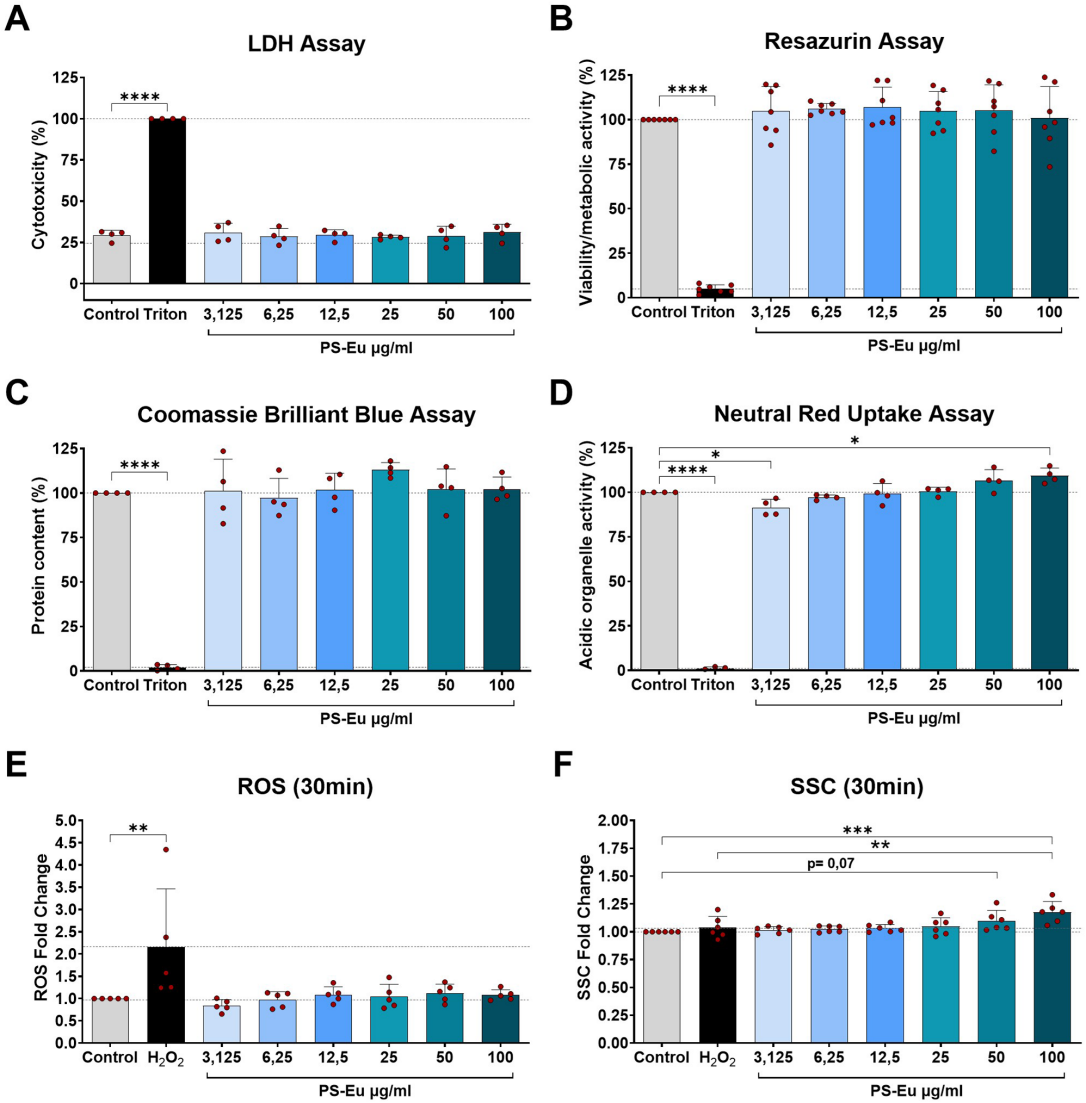
Impact of PS-Eu on A549 cells grown in submerged 2D conditions. Graphs represent the results obtained with **(A)** lactate dehydrogenase (LDH) assay (cytotoxicity), **(B)** Resazurin assay (cell viability/mitochondrial activity), **(C)** Coomassie brilliant blue (CBB) assay (protein content), **(D)** Neutral red uptake (NRU) assay (acidic organelles activity) of cells incubated for 48 h with increasing concentrations of particles. Additionally in **(E)** and **(F)** are shown reactive oxygen species (ROS) production, and cell inner granularity measurement (SSC), measured 30 min post treatment with flow cytometry. Statistical analysis was done using One-way ANOVA vs. untreated control. In SSC measurement, analysis was also performed vs. H_2_O_2_ control. All graphs show mean ± standard deviation and every dot represents an independent experiment. Abbreviations: A549: adenocarcinoma human alveolar basal epithelial cells; PS-Eu: europium doped polystyrene nanoparticles

After characterizing the particles used in this work, we tested their impact on A549 cells, an alveolar type II cell (ATII) model, using standard cytotoxicity tests and flow cytometry. In these tests, cells were grown for 24 h in submerged 2D culture conditions and then exposed to PS-Eu at different concentrations for 48 h. The results presented in Fig. 2 clearly indicate that neither lactate dehydrogenase (LDH) release nor mitochondria activity (Resazurin assay) or reactive oxygen species (ROS) production were affected in particle-treated samples compared to control cells, suggesting that they do not induce acute cytotoxicity (Fig. 2.A, B, E). Of note, the basal level of LDH detected in the samples can be ascribed to the presence of FBS in the CCM, which naturally contains LDH [39]. Similarly, no changes in protein content were seen using the Coomassie brilliant blue (CBB) assay (Fig. 2.C), thus we concluded that cells didn’t die or detach upon treatment in comparison to control cells. In contrast, a dose-dependent increase in acidic organelles activity was detected using the neutral red uptake (NRU) assay (Fig. 2.D). A similar trend was detected by flow cytometry in the side scatter channel (SSC) (Fig. 2F), which is indicative of cell granularity and is proportional to particle uptake [40]. Taken together, these data suggest that PS-Eu particles may interfere with the functionality of acidic organelles increasing their number, size or their activity, possibly as a consequence of particle uptake.

In these experiments, Triton X-100 and H_2_O_2_-treated samples successfully served as controls for minimum viability, minimum protein content and minimum acidic organelle activity, as well as the control for maximum LDH release and ROS production.

#### PS-Eu uptake is dose-dependent, and particles accumulate in endosomes and lamellar bodies under standard submerged 2D conditions

**Fig. 3:**
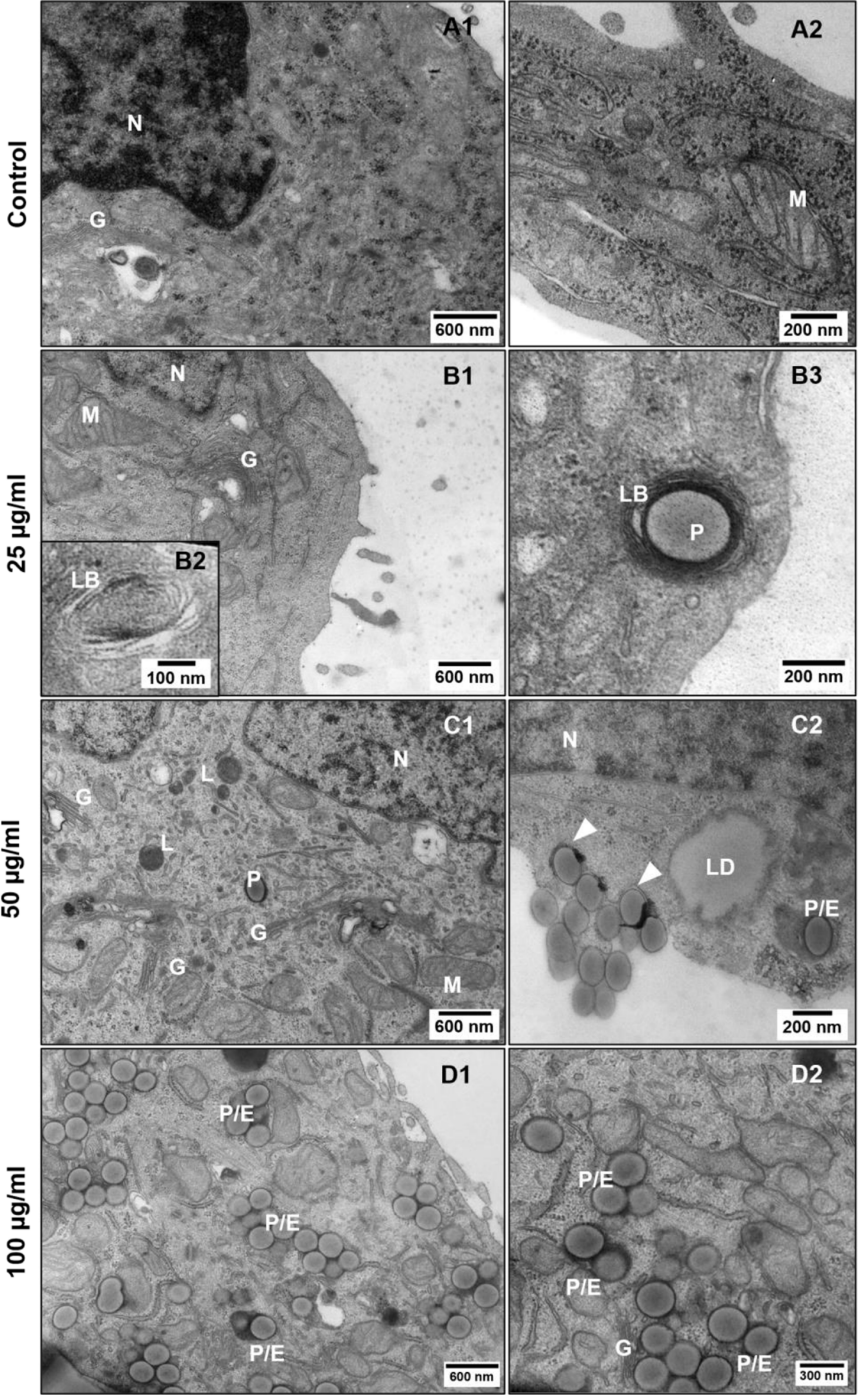
Particle internalization assessment in 2D grown A549 cells treated with increasing concentrations of nanoplastics. Control cells (**A1**-**2**), cells treated for 48 h with 25 µg/ml (**B1**-**3**), 50 µg/ml (**C1**-**2**) and 100 µg/ml (**D1**-**2**) PS-Eu. Abbreviations and legend: A549: adenocarcinoma human alveolar basal epithelial cells; LB, lamellar body; LD, lipid droplet; Arrowheads, plasma membrane foldings during particle internalization; G, Golgi apparatus; N, nucleus; P, PS-Eu particles; E, endosomes; L, lysosomes; M, mitochondria

Since PS is a polymer that is generally not biodegraded [41], we can assume that PS-Eu particles accumulate inside the cells, influencing the endo-lysosomal compartment functionality. To confirm the particle uptake and define cellular localization of particles, we performed TEM analyses on treated A549 cells grown under submerged 2D conditions and observed that the amount of the endocytosed particles was dose dependent. Particles were found within endosomes and occasionally in lamellar bodies (LBs) (Fig. 3.B3). The endosome membrane was strongly associated with the particle surface (Fig. 3.C2, arrows). No clear signs of apoptosis, such as chromatin condensation and apoptotic body formation, were detected in the treated cells. However, it is important to note that A549 cells, that were grown in submerged conditions for a short period of time, are poor in LBs and have very few microvilli (Fig. 3.B1-B3, D1) or cell-cell junctions, which suggests that they are not yet terminally differentiated into an ATII phenotype and do not yet form a functional epithelial barrier.

Thus, our future experiments were performed using a 3D culture model, optimized within this study, that uses transwells and quasi air-liquid interface (ALI) culture conditions to better mimic the alveolar microenvironment. To assess the importance of quasi-ALI conditions for cell-particle interactions, we also cultured the cells in transwells under submerged 3D conditions (Sub) and used them for a comparison, as described in following sections.

#### 3D-grown A549 cells successfully form an epithelial barrier and strongly resemble ATII cells

**Fig. 4:**
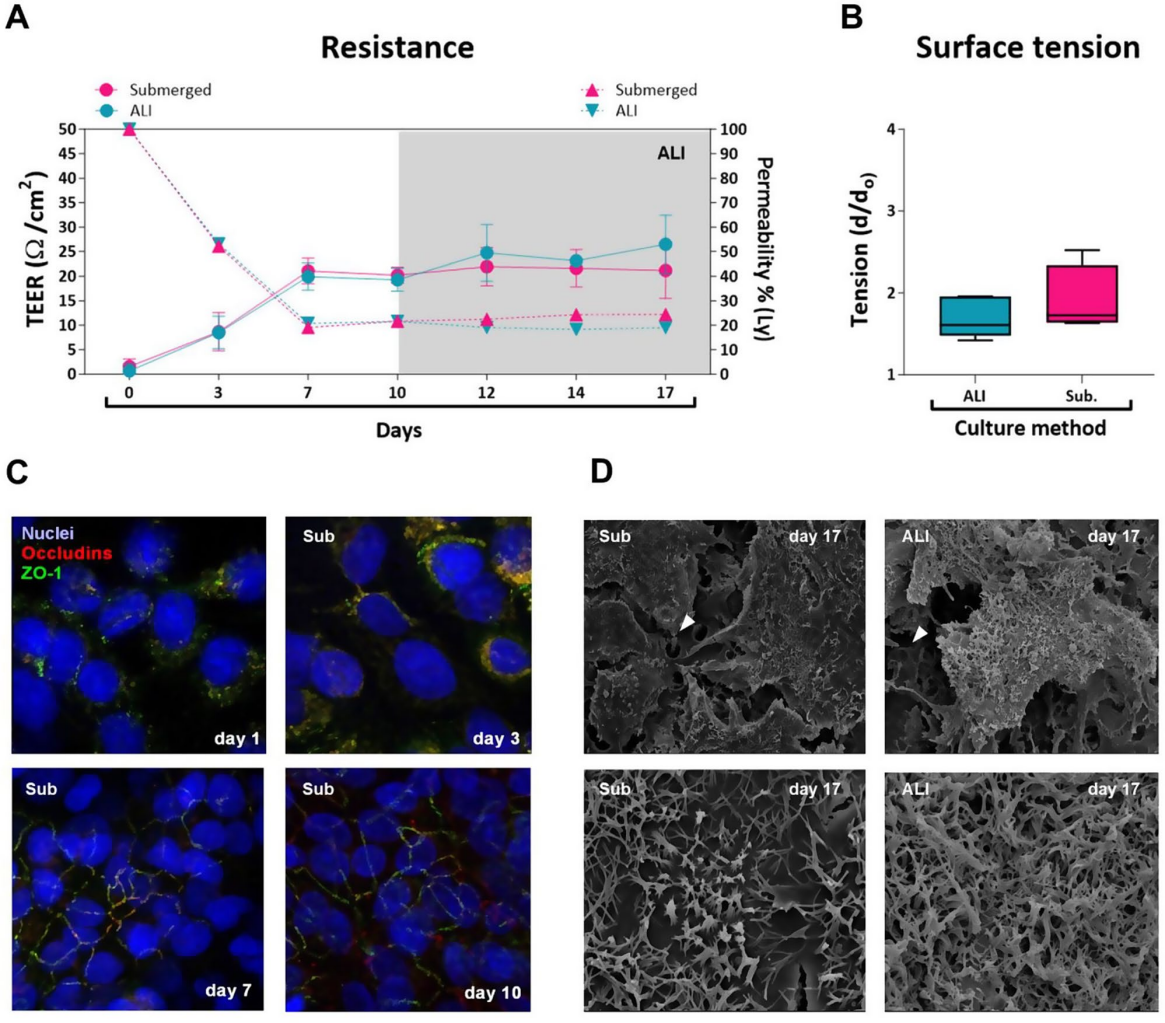
3D model characterization. **(A)** graph depicting over time formation of the lung alveolar barrier, in terms of transepithelial electrical resistance (TEER, solid line), and permeability measured with lucifer yellow assay (Ly) (dashed line) in quasi-air-liquid interface (ALI, blue) and submerged (pink) cultures; **(B)** graph depicting the surface tension of the epithelial barrier estimated with the drop-spreading method; **(C)** Confocal microscopy of adenocarcinoma human alveolar basal epithelial cells (A549) grown for different periods in the transwells in submerged condition. Blue: DAPI (nuclei), red: occludins, green: zonula oclludens-1 (ZO-1); magnification 1000x. **(D)** Scanning electron microscopy images of cells grown for 17 days in the transwells, (left) cells grown in submerged conditions, (right) cells grown in quasi-ALI conditions. Magnification top figures: x2500, bottom figures: x10000. Arrowheads indicate the membrane pores. Sub: submerged; d: diameter

After excluding that PS particles do not cause acute cytotoxicity, we developed a more biologically relevant model using transwells. Here, A549 cells were seeded in transwells and grown under submerged conditions until they formed an epithelial barrier characterized by an intricate net of tight junctions. Then, half of the samples were kept in submerged (Sub) conditions or let air-exposed (quasi air-liquid interface (ALI) conditions) for an additional 7 days before being treated with the particles. Since in quasi-ALI conditions cells are exposed to air, that represents more real-life conditions, we expected a more intensive “differentiation” of A549 towards an ATII phenotype [42–45]. To determine the best time to induce quasi-ALI and define when the epithelial barrier is established, we monitored its formation by measuring both transepithelial electrical resistance (TEER) and epithelial permeability over time (OT) using the EVOM3 and the lucifer yellow (Ly) assay. The results show that the epithelial permeability decreased over time until the curve reached a plateau on the 7th and 10th day after seeding. Similar results were obtained with the TEER measurements, which increased until reaching, in agreement with the literature, stable values of 20–25 Ohms/cm^2^ during the same period (Fig. 4.A), suggesting the formation of a continuous A549 epithelium [46]. To confirm this hypothesis, we performed an OT confocal microscopy experiment where cells, after being grown in transwells for different time periods, were stained with fluorescent antibodies directed against occludins and zonula occludens-1 (ZO-1) proteins. The results obtained confirmed our previous findings and showed that such proteins successfully assemble to form an intricate net of tight junctions on day 7 and 10 (Fig. 4.C). We then followed the cells for an additional 7 days and observed no differences in TEER or permeability between the different culture systems. On day 17th after seeding, we additionally examined both the surface tension, with the drop spreading method, and cell morphology with SEM in both samples (Fig. 4.B and D). The results indicate that quasi-ALI cells produce more surfactants (lower tension) and have different surface morphology, rich in protrusions, when compared to submerged cultures, suggesting that they may more closely resemble ATII cells as described by Fehrenbach in 2001 [47]. A schematic representation of the described model can be found in Additional File 2 together with some transwells sections of control cells grown in quasi-ALI or submerged conditions.

#### PS-Eu decrease surface tension in A549 cells under quasi-ALI and submerged conditions

**Fig. 5:**
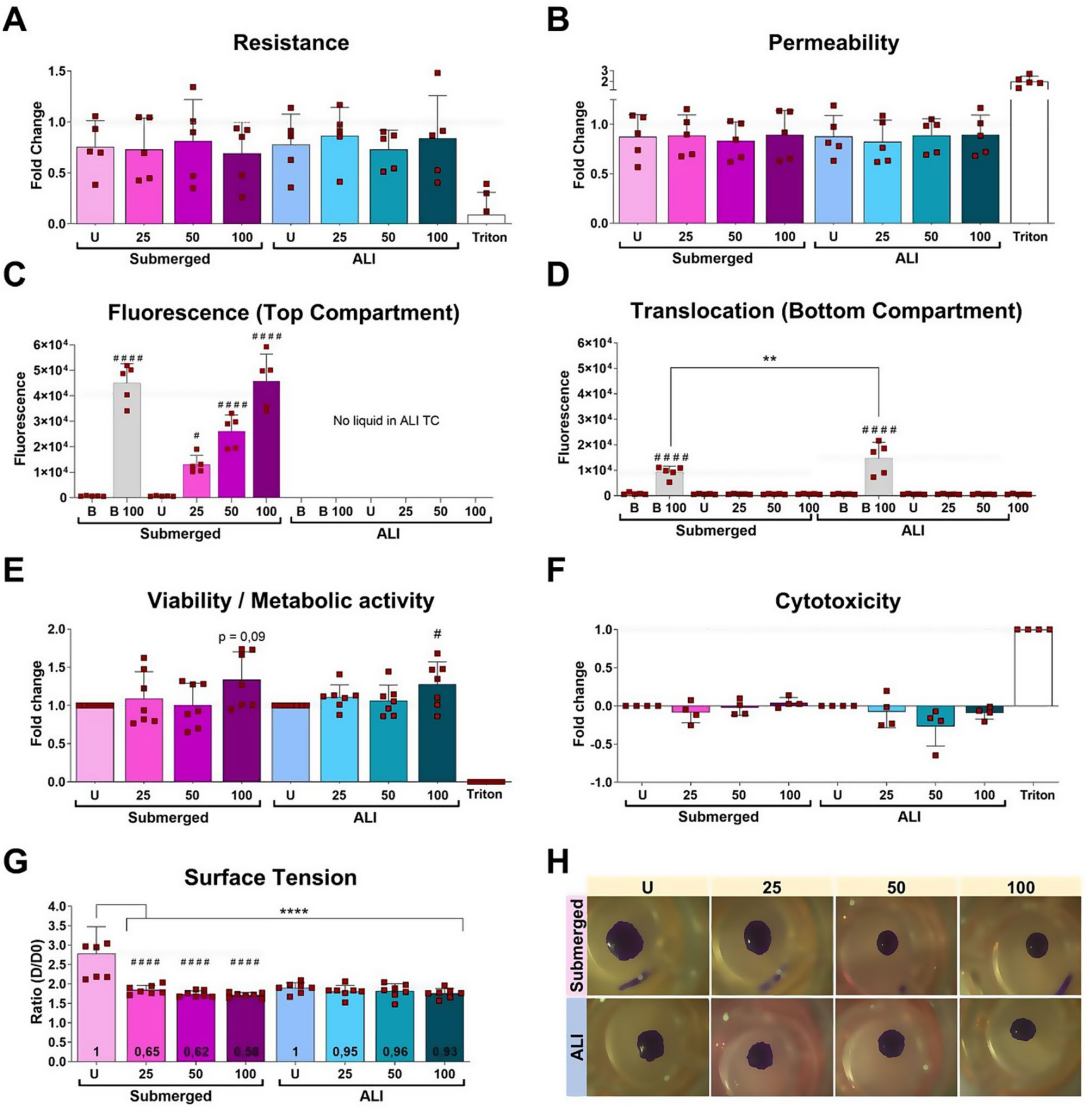
Impact of PS-Eu on A549 cells grown on transwells (3D). After incubating cells with increasing concentration of PS-Eu (25, 50 and 100 µg/ml) the following parameters were measured. **(A – B)** Impact on epithelial barrier formation in terms of resistance (transepithelial electrical resistance (TEER)) and epithelial permeability (quantified by lucifer yellow (Ly) assay) measured in cells grown either in submerged (pink/purple) or quasi-air-liquid interface (ALI) conditions (blue). Data are presented as fold changes from the values measured prior treatment. **(C-D)** Particle translocation measured by detecting Eu fluorescence in the bottom compartment **(D)** of the transwell. The fluorescence in the top compartment **(C)** was measured to confirm particle presence after treatment. B, negative control, cell-free transwells. B 100, positive control for translocation, a cell-free transwells treated with 100 µg/ml PS-Eu. **(E-F)** Effect of PS-Eu particles on cell viability/metabolic activity (measured by Resazurin assay) and their cytotoxic potential (measured by LDH assay). Data are presented as fold change vs. the positive controls (U, for Resazurin assay, and Triton, for LDH assay). Significance was calculated using One-way ANOVA vs. the respective untreated controls (#). Significance vs. Triton is not shown. **(G)** PS-Eu particle impact on the surface tension of the epithelial barrier. Statistical analysis was performed either using One-way ANOVA on particle-treated samples vs. the U controls (#), or with a Two-way ANOVA vs. all samples (*). The numbers enclosed in the bars represent the fold change vs. the correspondent U control. All graphs in Fig. 4 show mean ± standard deviation and every dot represents an independent experiment. **(H)** representative images obtained during the drop-spreading experiment. Ly: lucifer yellow; D: diameter; U: untreated; LDH: lactate dehydrogenase

After characterizing and developing the quasi-ALI model, we assessed the impact of PS-Eu particles on transwells-grown A549 cells cultured both in quasi-ALI and submerged 3D culture condition (Sub) applying the same mass of nanoplastics. The data obtained showed that no significant changes between particle-treated and control samples were observed in terms of epithelial barrier integrity (transepithelial electrical resistance and Ly permeability) (Fig. 5A-B). Additionally, no particle translocation across epithelial barrier/epithelial layer was detected as shown in Fig. 5.C-D. In the apical media, the detected fluorescence signal was correlated with particle exposure doses.

We detected no cytotoxicity in any of the PS-Eu-treated samples when compared to controls, however, we found a significantly increased viability/metabolic activity in quasi-ALI cultures incubated with 100 µg/ml of particles. A similar, but not-significant trend was also visible in submerged 3D cultures. This is in contrast with the data obtained in short-grown 2D submerged cultures, where no changes were detected, however, we cannot exclude that the cells, after such a long culture time, can be subjected to structural changes to adapt to the new conditions and thus respond differently to treatment (Fig. 5.E-F). In both barrier functionality and viability assessments, Triton X-100 successfully served as a control for minimum viability and resistance and maximum permeability and cytotoxicity, as it is known to cause cell death and dissolution.

Then we assessed surface tension (ST), a cell-specific biomarker, using the drop spreading method and found a significant difference between control cells grown in submerged versus quasi-ALI settings (Fig. 5.G), showing the positive impact of quasi-ALI conditions on surfactants production. PS-Eu instead, were able to significantly decrease ST in comparison to controls in submerged samples. In contrast, despite not statistically significant effects were also seen in quasi-ALI samples we observed a constant decrease of ST in response to treatment, suggesting that particle can promote the release of surfactants, but that this effect is milder compared to submerged cells, possibly because this process is already conducted a full regime in air-exposed cells (Fig. 5.G). Figure 5.H shows some representative images collected during the experiment, demonstrating how the diameter of the deposited drops varies depending on the type of treatment the cells were exposed to.

Ultrastructure analysis might provide more insight into defining why particles can affect surface tension and metabolic activity in treated cells.

#### PS-Eu localize in endosomal vesicles and lamellar bodies affecting surfactant production under quasi-ALI and submerged 3D conditions

To define PS-Eu cellular localization and assess if particle exposure can cause ultrastructural changes in transwells-grown A549 cells, we inspected treated quasi-ALI and submerged samples via TEM.

**Fig. 6:**
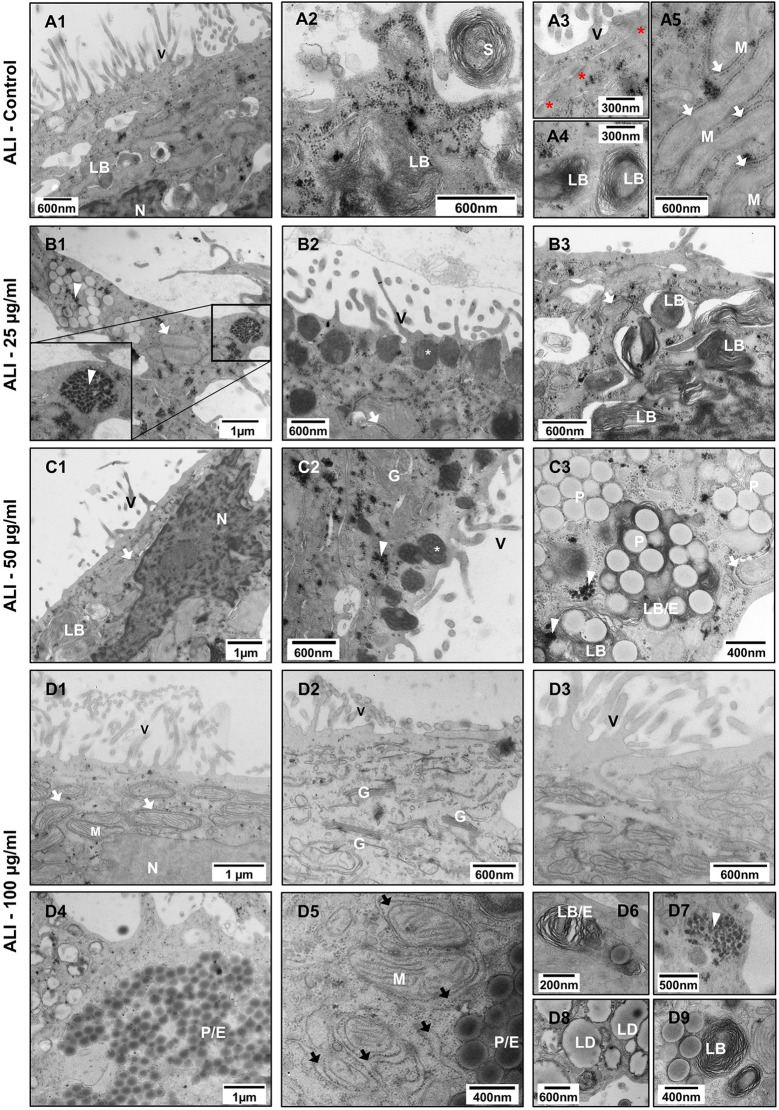
Ultrastructural changes in quasi-ALI A549 cultures treated with increasing particle concentrations. Transmission electron microscopy images showing untreated control cells **(A1-5**) and cells treated with 25 (**B1-3**), 50 (**C1-3**) and 100 µg/ml (**D1-9**) of PS-Eu. Abbreviations and legend: ALI, quasi-air-liquid interface; A549: adenocarcinoma human alveolar basal epithelial cells; LB, lamellar body; LD, lipid droplet; E, endosomes at different stages of maturation; Arrowhead, aggregate; Arrows, mitochondria associated ER membranes (MAMs); Black arrow, dilated ER cisternae; N, nucleus; P, PS-Eu particles; M, mitochondrion; V, microvilli; S, surfactant; G, Golgi apparatus; White asterisk, secretory lysosome; Red asterisk, tight junctions

As shown in Fig. 6.A1-A5, quasi-ALI cultures better resembled lung ATII cells. In fact, they were rich in lamellar bodies (LBs), microvilli (V) and secrete surfactants into the extracellular fluid (S). These cells were structurally and functionally more differentiated than the cells shown in Fig. 3, indicating that quasi-ALI conditions are important for cell differentiation and specialization. Additionally, these cells were rich in mitochondria-associated ER membranes (MAMs), which are known to play an important role in the synthesis of lipid surfactant components such as phosphatidylcholine (PC) and phosphatidylglycerol (PG) (Fig. 6.A5) [48]. Our results are partly in contrast to other publications. In fact, despite the occasional presence of localized multiple cell layers, we did not see superficial cell necrosis after 14 days of culture as shown by Meindl et al., possibly because we used transwells with larger pores and cells had easier access to the nutrients in the basolateral compartment [49].

**Fig. 7:**
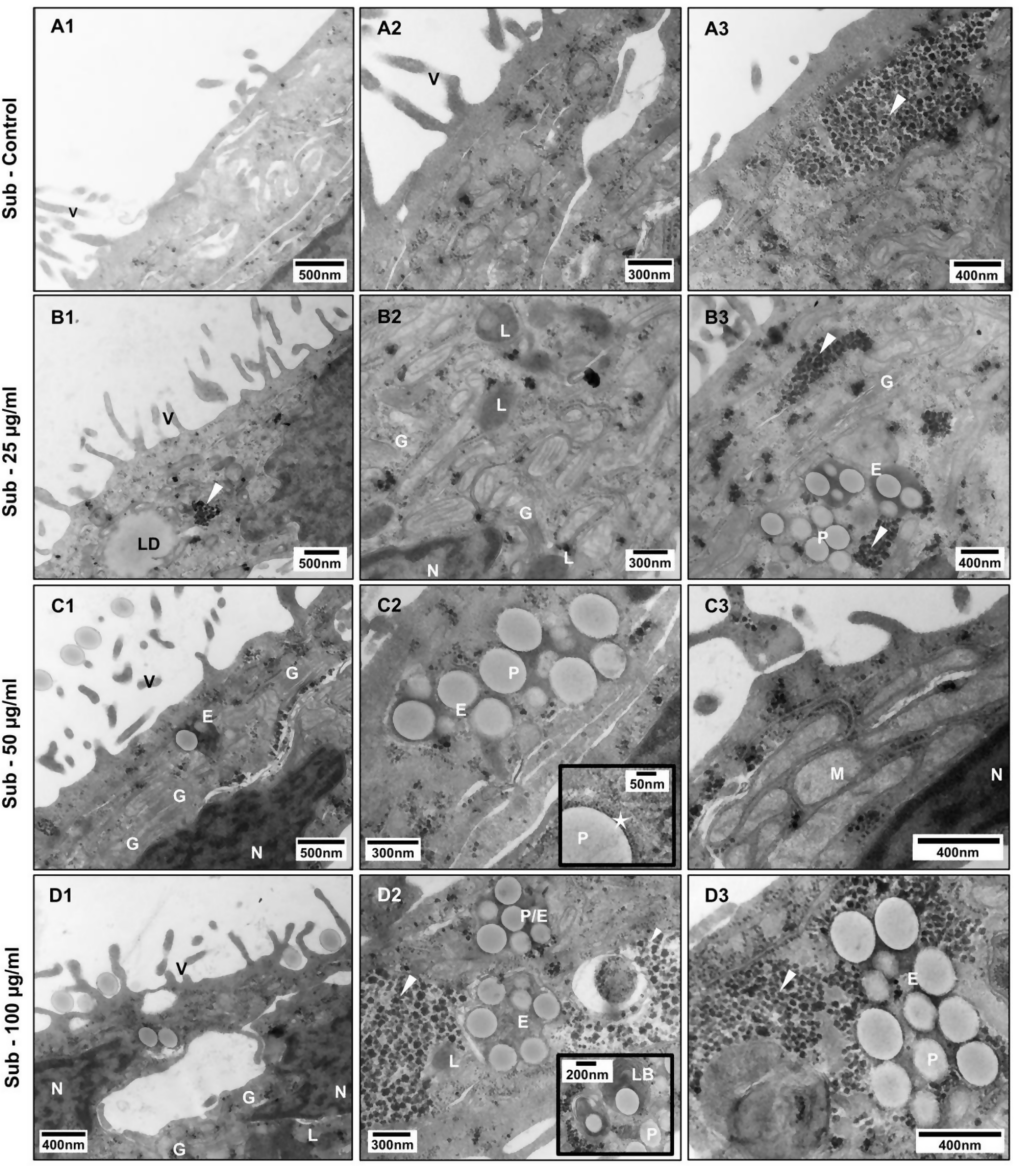
Ultrastructural changes in submerged-grown A549 cells treated with increasing particle concentrations. Transmission electron microscopy images showing untreated control cells (**A1**-**3**), and cells treated with 25 (**B1**-**3**), 50 (**C1**-**3**) and 100 µg/ml (**D1**-**3**) of PS-Eu. Abbreviations and legend: A549: adenocarcinoma human alveolar basal epithelial cells; LB, lamellar body; LD, lipid droplet; L, lysosome; E, endosomes at different stages of maturation; G, Golgi apparatus; arrowhead, aggregate; arrows, mitochondria associated ER membranes (MAMs); star, membrane tightly surrounding a nanoparticle; N, nucleus; P, PS-Eu particles; M, mitochondrion; V, microvilli

In submerged cells instead, we detected shorter and sparser microvilli (Fig. 7.A1), rare lamellar bodies and MAMs, as well as some intracellular aggregates. These are found also in control samples although in lower amounts when compared to treated submerged cells.

When particles are applied to quasi-ALI cultures, we see a dose-dependent increase in particle uptake with a reduction in the amount of LBs (which look immature due to the presence of inclusions) coupled with the appearance of the same aggregates found in submerged 3D cultures (Fig. 6.B1, C3 and D7) which strongly resemble rosette glycogen described by Stahlman et al. in surfactant protein B (SP-B) +/- mouse models [50]. These findings might suggest that particle treatment could cause an alteration of SP-B production. At particle concentrations of 25 µg/ml and 50 µg/ml, we occasionally see the presence of electron-dense vesicles close to the cell surface resembling secretory lysosomes (Fig. 6.B2 and C2), which might be involved in plasmalemma remodelling to counteract membrane loss induced by intense particle uptake [51]. Notably, in 100 µg/ml-treated cells, we found particles in both LBs and autophagosome/endosomes-containing LBs (Fig. 6.D4, D6 and D9), as well as a marked reduction in MAMs, often characterized by the presence of dilated cisternae (Fig. 6.D5), a hallmark of ER-stress. Occasionally, cells with several lipid droplets (LD) were also found. Interestingly, particle uptake appeared to be not only dose-dependent but also cell-dependent. Indeed, particle-free cells with normal ultrastructure (Fig. 6.D1-D3) were found surrounded by cells fully loaded with particles and characterized by the above-mentioned ultrastructural changes (Fig. 6.D4). This could indicate that particle uptake induces this kind of altered phenotype.

Instead, in treated submerged 3D samples, we observed a further reduction in the number of MAMs (which were already rare in these cells) compared to controls. More lysosomes were found distributed throughout the cell and not only in the apical cytoplasm close to the cell surface, suggesting that they are probably non secretory. In addition, many Golgi stacks (Fig. 7.D1) as well as dilated ER cisternae and some mitochondria with distinctly fewer cristae (Fig. 7.C3) were present in these cells. Again, particles were mainly found in endosomes or autophagosomes and rarely inside LBs (Fig. 7.D2), which are only occasionally present in submerged cells. In both culture models, particles were found surrounded by a tightly adherent membrane as shown in Fig. 7.C2, star. Interestingly, most of the above ultrastructural changes could not be predicted by either standard cytotoxicity tests or barrier integrity assessment, further evidencing the importance of examining cell ultrastructure when conducting toxicological studies.

Taken together, these results indicate that endocytosed particles can trigger a stress response that is reflected in altered cell ultrastructure, ultimately leading to a reduction in the number of LBs and MAMs while increasing the number of particle-filled digestive/autophagic compartments and LBs undergoing degradation.

**Fig. 8:**
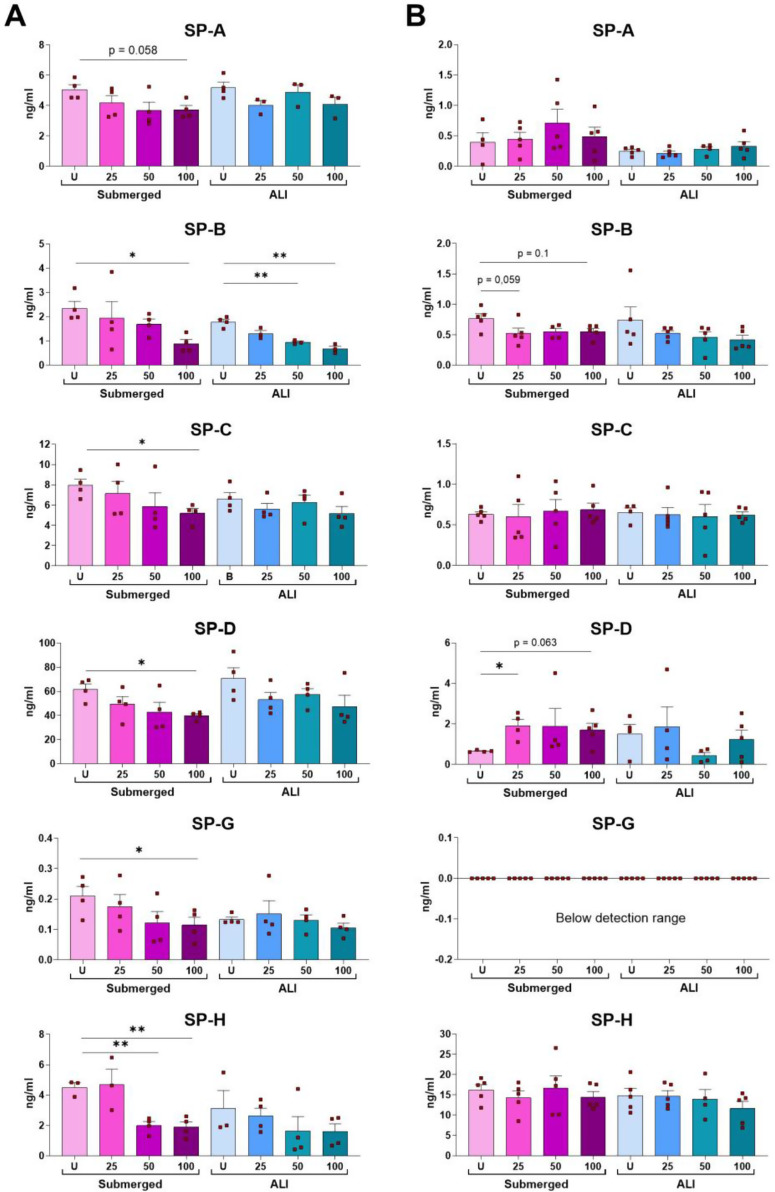
Impact of PS-Eu particles (25, 50 and 100 µg/ml) on surfactant protein (SP) production. Surfactant proteins were quantified via ELISA performed on **(A)** the cell pellet (intracellular fraction) or **(B)** cell supernatant (secreted fraction) of 48 h-treated adenocarcinoma human alveolar basal epithelial cells (A549) grown either in quasi-air-liquid interface (ALI) or 3D submerged conditions. Statistical analysis was performed using One-way ANOVA on particle-treated samples vs. the untreated (U) controls. Graphs show mean ± standard deviation and each dot represents an independent experiment

Since MAMs and LBs are structures critically involved in surfactant production and secretion, we then quantified the amount of surfactant proteins (SP), a specific cell-function biomarker, both within cells (cell pellet) (Fig. 8.A) and in the supernatant (Fig. 8.B) of our samples via ELISA.

The results revealed that all the surfactant proteins investigated (SP-A, -B, -C, -D, -G, -H) were produced by untreated cells (pellet) both in quasi-ALI and submerged 3D conditions, however, SP-G was undetectable in the extracellular fraction. Interestingly, particle treatment promoted a dose-dependent reduction trend in the intracellular SP production in both culture models, but this occurred only marginally in the extracellular pool where only SP-B and SP-D secretion were influenced exclusively in submerged samples.

### Impact of PS-Eu on human bronchial model

#### PS-Eu do not change metabolic activity or the barrier integrity of quasi-ALI Calu-3 cultures

**Fig. 9:**
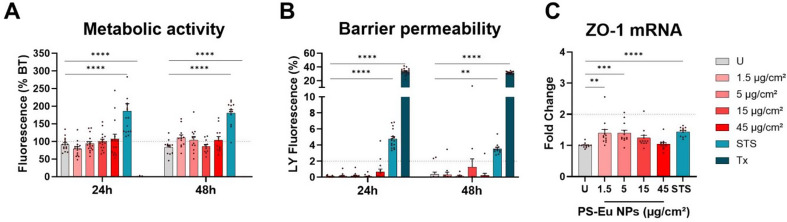
Cytotoxicity of PS-Eu on quasi-ALI Calu-3 cultures. Cultured human airway epithelial cells (Calu-3) were grown in transwells in quasi-air-liquid interface (ALI) conditions for 7 days before 24 h and 48 h of treatment with droplets of europium doped polystyrene nanoparticles (PS-Eu) (from 1.5 to 45 µg/cm^2^). Staurosporine (STS) at 0.75 µM and Triton X-100 (Tx) at 0.01% were used as positive controls. The metabolic activity of the cultures **(A)** was evaluated by adding Alamar Blue (AB) to the basal compartment and quantifying its metabolization after 2 h by spectrofluorimetry. Results are expressed as % of the metabolic activity measured before treatment (BT) for each culture (dotted line). The barrier integrity **(B)** was evaluated by adding lucifer yellow (Ly) to the apical compartment, and the % of translocation to the basal compartment was quantified after 1 h by spectrofluorimetry. Gene expression analysis of zonula occludens-1 (ZO-1) mRNA **(C)** performed using ubiquitin C (UBC) and hypoxanthine phosphoribosyl transferase (HPRT) as reference genes. Results are expressed as fold change of untreated control (U). 3 independent experiments with 4 **(A**, **B)** or 3 **(C)** technical replicates were performed and reported as mean ± standard error of mean. Statistical analysis was performed with two-way ANOVA in **A** and **B**, and one-way Anova in **C**

To study the impact of PS-Eu on the bronchial epithelium, we used the Calu-3 cell line cultured on 3 μm pore transwells at ALI for 7 days before treating them with droplets of PS-Eu suspensions to maintain a quasi-ALI condition. Effects of PS-Eu on the metabolic activity and barrier integrity were evaluated by AlamarBlue and lucifer yellow (Ly) assays respectively. Exposure to PS-Eu at different concentrations (1.5–45 µg/cm²) did not exhibit a significate modulation of the metabolic activity at 24 h and 48 h of exposure in contrast to the positive controls staurosporine (STS) (0.75µM) that increased metabolic activity while Triton (Tx) treatment (at 0.01%) completely abolished the metabolic activity of the Calu-3 cell cultures (Fig. 9.A). Barrier integrity assessed by measuring its permeability to Ly confirmed that, in line with A549 experiments, no loss of barrier integrity was observed at the tested concentrations. The positive controls staurosporine and Triton altered the barrier integrity as the % of Ly reaching the basal compartment was above the value of 2% established as the limit for Calu-3 cultures (Fig. 9.B) [52]. We also quantified mRNA expression of the tight-junction associated protein zonula occludens-1 (ZO-1) at 24 h of exposure to PS-Eu. Results show that this mRNA was even slightly increased by PS-Eu exposure at the two lowest concentrations tested (1.5 and 5 µg/cm^2^, Fig. 9.**C**) pointing to an adaptive response to maintain the barrier integrity. Though, the positive control staurosporine also upregulated the ZO-1 mRNA expression.

Altogether these results suggest that particles do not reduce barrier integrity over the tested period and do not affect cytotoxicity-related biomarkers.

#### PS-Eu particles are taken up by Calu-3 cells in submerged 2D and quasi-ALI 3D cultures but do not translocate through the bronchial barrier

**Fig. 10:**
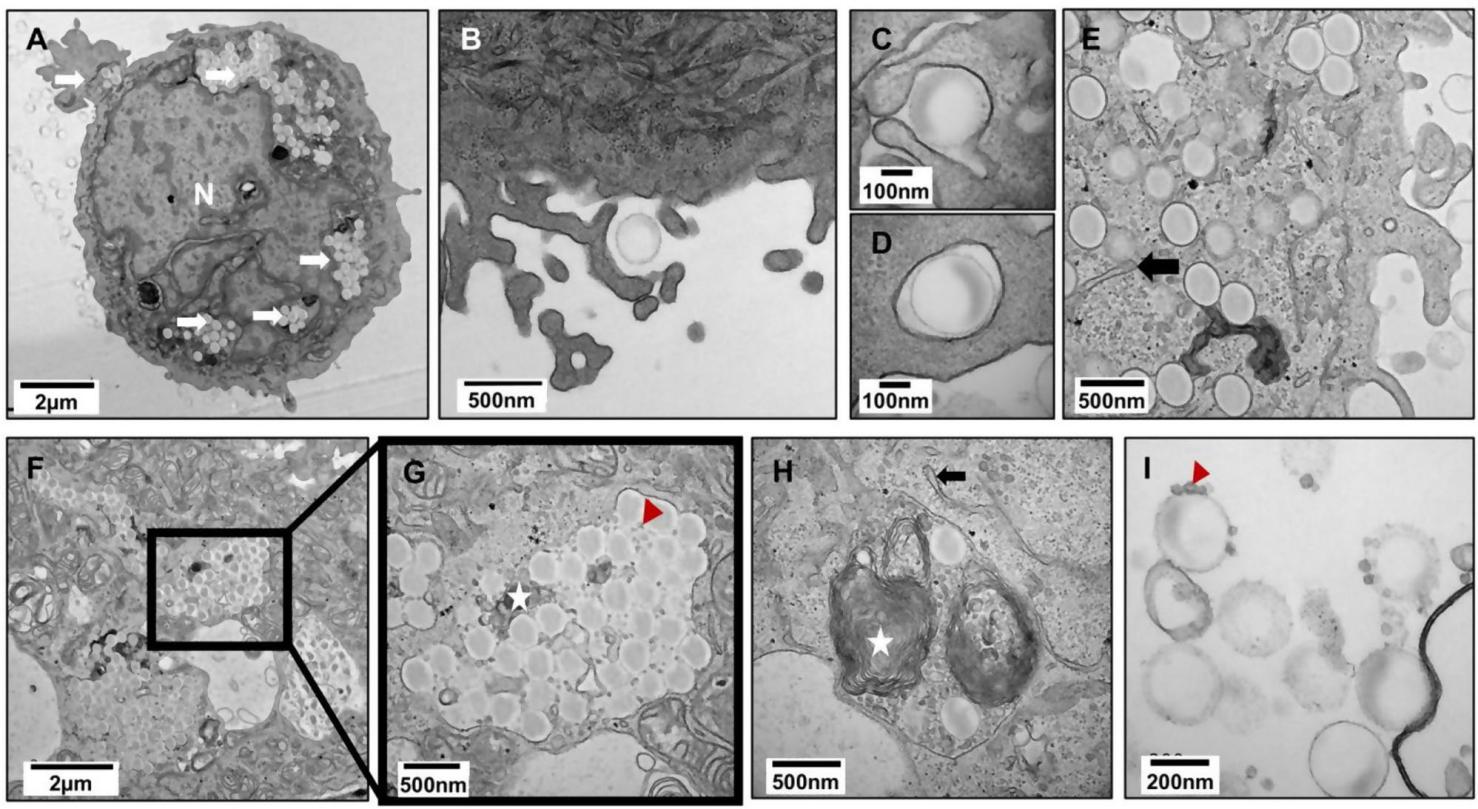
Particle localization in 2D grown Calu-3 cells. Cultured human airway epithelial cells (Calu-3) were exposed to 45 µg/cm^2^ of europium doped polystyrene nanoparticles (PS-Eu) for 24 h and then images via transmission electron microscopy. **(A)** A cell fully loaded with PS-Eu particles. **(B-D)** Three successive stages of particle internalization. **(E)** Numerous PS-Eu individually distributed in the apical cytoplasm with membrane adherent to the particle surface. **(F-G)** Large particle-containing vesicles in the interior of the cell. **(H)** Autophagosome with PS-Eu particles, LBs and glycogen-like granules. **(I)** Granules adhering on particle surface and PS-Eu particles interacting with membrane debris in the extracellular space. Legend: white arrow: PS-Eu particles; N: Nucleus; black arrow: dilated cisterna of endoplasmic reticulum; white star: lamellar body (LB); red arrowhead: glycogen resembling-granules

**Fig. 11:**
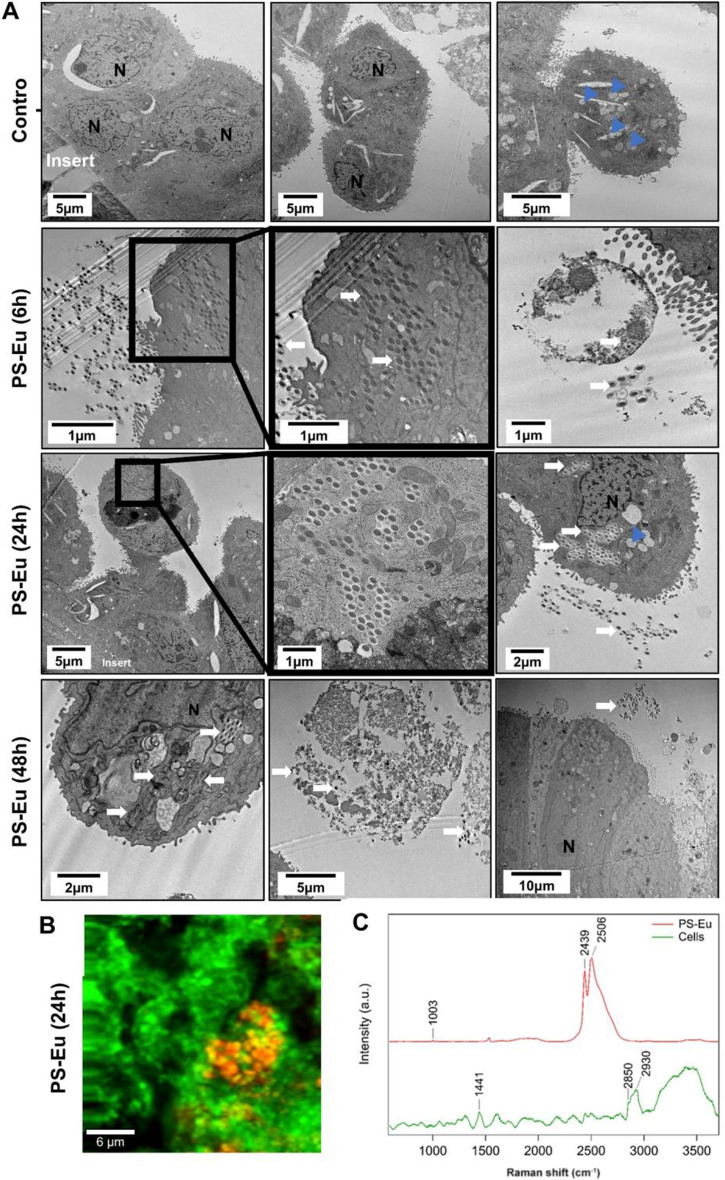
Particle localization in quasi-ALI Calu-3 cultures. **(A)** Transmission electron microscopy images of cells exposed to 45 µg/cm^2^ of europium doped polystyrene (PS) nanoparticles (PS-Eu) for 6 h, 24 h and 48 h under quasi-air-liquid interface conditions (ALI). White arrow, PS-Eu; blue arrowhead, mucus vesicles. **(B-C)** Detection of PS-Eu in the epithelium by Raman microscopy. **(B)** Combined Raman image of cultured human airway epithelial cells (Calu-3) exposed to PS-Eu for 24 h. The Raman image of cells (green) and PS-Eu (red) was taken at z = 7 μm above the transwell. **(C)** Raman spectrum of PS-Eu measured inside the epithelium. The Raman bands specific to PS (1001 cm-1), Eu (2440 and 2504 cm-1), and proteins and lipids (1441, 2850, 2930 cm-1) are indicated

To investigate PS-Eu internalization in Calu-3 cells, we examined their uptake in 2D (submerged) and 3D (quasi-ALI) cultured Calu-3 cells by TEM. Calu-3 cells were exposed to 45 µg/cm^2^ PS-Eu for 24 h for 2D cultures (Fig. 10) and at different time points for 3D cultures (6 h, 24 h and 48 h) (Fig. 11).

In 2D cultured Calu-3 cells, TEM analysis showed, like A549 cells, that PS-Eu interacted closely with cell membranes and were taken up individually by forming endosomes/phagosomes whose membranes tightly surrounded the particle (Fig. 10.A-E). However, since PS-Eu were also seen extracellularly attached to membrane debris (Fig. 10.I) we can suggest that such contacts might be promoted by hydrophobic interactions rather than receptor mediated processes. TEM analysis revealed that PS-Eu particles were massively internalized in 2D cultures and, once they reached the perinuclear area, they aggregated in big phagosomes (Fig. 10.F-G) which contained also LBs (white star) and glycogen-like granules (red arrow) (Fig. 10.H). Such granules were also found extracellularly, attached to the surface of particles possibly as result of cell death, or exocytosis (Fig. 10.I). Similarly to A549 cells, Calu-3 cells exhibited an enlarged and dilated endoplasmic reticulum which is indicative of ER stress when exposed to PS-Eu (Fig. 10.E).

In quasi-ALI cultured Calu-3 cells, the majority of PS-Eu were observed at the apical surface of the culture, suggesting that some particles may be trapped in the mucus layer produced by Calu-3 cells. PS-Eu internalization was more prominently observed at 6 h and 24 h compared to 48 h. In fact, at 48 h, particle uptake was only observed in a few superficial round-shaped cells, which might suggest that cells that have internalized PS-Eu slowly detach from the pseudostratified epithelium after some hours of treatment (Fig. 11.A). These superficial cells were still attached to the epithelial monolayer as they were not washed off during the rinsing steps of TEM preparation. The interaction of extracellular PS-Eu with membrane debris and granules (Fig. 10.I) which were also observed in intracellular vesicles containing PS-Eu (Fig. 10.G-H) supports this conclusion on detachment of cells after particle internalization. The round shape and location of the cells at the top of the epithelial sheet may expose a larger cell surface area to the particles, making them more likely to internalize particles (Fig. 11.A). A similar particle distribution was seen in A549, where apical cells were found to internalize more particles when compared to deeper cells. These rounded cells were also present in control Calu-3 cultures.

Compared to the submerged 2D cultures, quasi-ALI cells presented a lower number of internalized particles possibly due to their smaller apical cell surface and increased mucin production, which may form a protective layer that prevents particle uptake. Interestingly, PS-Eu, upon internalization, were found associated with LBs but not with mucus vesicles, possibly because their intracellular trafficking is different from LBs as mucus is not subjected to a recycling process that can favour particle-mucus co-localization/ingestion.

These observations were confirmed by Raman microscopy on quasi-ALI cultures (Fig. 11.B-C and Additional File 3). Using the specific Raman bands of polystyrene (1001 cm^− 1^), Europium (2440 and 2504 cm^− 1^), and biomolecules (proteins at 1441 and lipids at 2850 and 2930 cm^− 1^), we identified internalized PS-Eu NPs in the bronchial epithelium (5 to 10 μm above the transwell membrane). The signal of Eu and PS co-localized indicating that the Eu did not leak. We also observed that not all cells internalized PS-Eu particles, and that some cells internalized several particles. This could suggest that some cells are more prone to uptake PS-Eu particles, as observed by TEM.

**Fig. 12:**
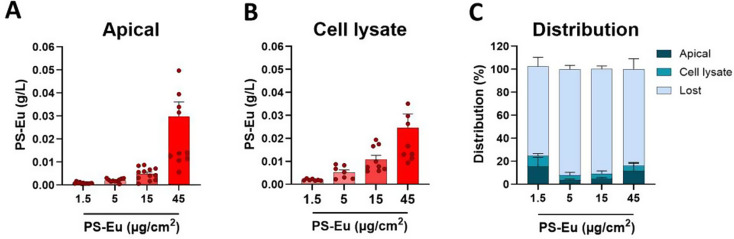
Quantification of PS-Eu in quasi-ALI Calu-3 cultures by fluorescence spectroscopy. Cells were exposed to 1.5–45 µg/cm² of PS-Eu particles for 48 h and then PS-Eu fluorescence was quantified in the compartment apical **(A)** and basal (not shown) as well as in the cell lysate **(B)** by fluorescence spectroscopy at λ_Ex_ = 264 nm. Distribution percentages were calculated relatively to the quantity of PS-Eu used for exposure **(C)**. Cells were lysed using milliQ water. Data are reported as mean ± standard error of mean. Abbreviations: quasi-air-liquid interface conditions (ALI); cultured human airway epithelial cells (Calu-3)

To quantify the distribution of PS-Eu in the bronchial 3D model, PS-Eu were quantified by measuring Eu fluorescence both in the apical and basal medium of the transwells, and in cell lysate after 48 h of exposure (Fig. 12.A-B). In the apical media, PS-Eu concentrations were correlated with exposure doses. The maximum percentage of NPs that was estimated in the apical compartment was 24.22%. PS-Eu concentrations in the cell lysate increased in a dose-dependent manner, confirming internalization observed by TEM and Raman microscopy. It is important to note that cell lysate represents intracellular NPs and extracellular NPs interacting with the cells, that were not removed by apical rinsing. A maximum percentage of 12.88% was detected in the cell lysates. Similarly to A549, PS-Eu were not detected in the basal medium suggesting that particles were not translocated or were translocated but got trapped in transwell pores or adsorbed on wells surfaces. This might also explain distribution percentages that showed that at least 67.4% of PS-Eu were not detected (Fig. 12.C).

#### Exposure to PS-Eu induced a slight pro-inflammatory response and mucins production without inducing an antioxidant response in quasi-ALI Calu-3 cultures

**Fig. 13:**
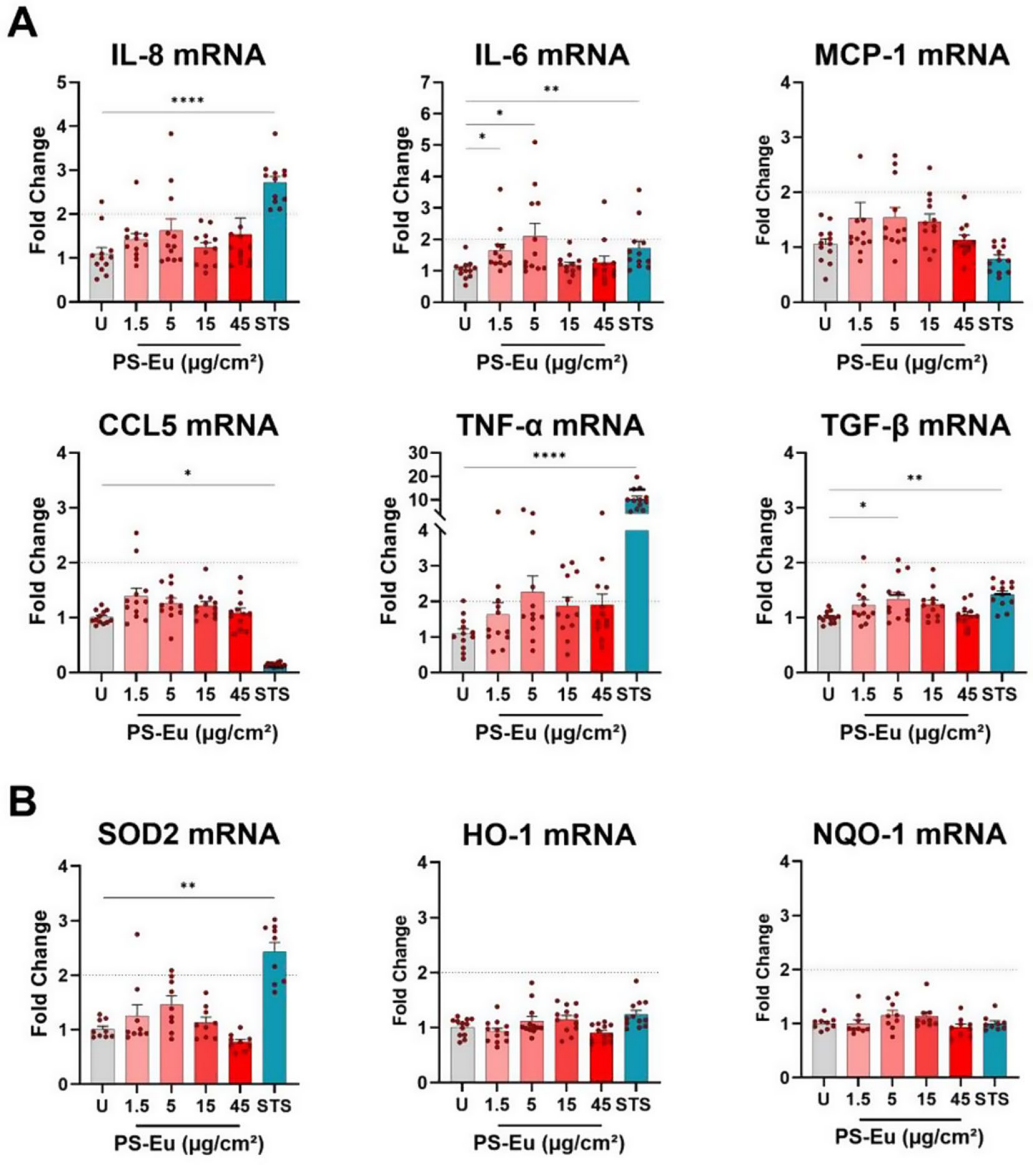
Cytokine, chemokine and antioxidant gene expression in quasi-ALI Calu-3 cultures after treatment with PS-Eu. Calu-3 cells were cultured in quasi-ALI conditions for 7 days before 24 h treatment with droplets (18 µl/cm^2^) of PS-Eu. Particles were applied from 1.5 to 45 µg/cm^2^ and 0.75 µM staurosporine (STS) was used as positive control. After exposure, RT-qPCR was performed using UBC and HPRT as reference genes. Soluble mediators (IL-8, IL-6, TNF-α, MCP-1, CCL5 and TGF-β) **(A)**, and oxidative stress markers (SOD2, HO-1 and NQO1) **(B)** mRNA expression is shown as fold change of control. 3 independent experiments with 3 technical replicates were performed and reported as mean ± standard error of mean. Statistical analysis was performed with one-way ANOVA. Abbreviations: mRNA: messenger ribonucleic acid; ALI: quasi-air-liquid interface conditions; Calu-3: cultured human airway epithelial cells; PS-Eu, europium doped polystyrene nanoparticles; IL-8: interleukin 8; IL-6: interleukin 6; MCP-1: monocyte chemoattractant protein-1; CCL5: chemokine (C-C motif) ligand 5; TNF-α: tumor necrosis factor-α; TGF-β: transforming growth factor-β; SOD2: superoxide dismutase 2, HO-1: heme-oxygenase 1 (HO-1); NQO-1: NAD(P)H quinone dehydrogenase 1; HPRT: hypoxanthine guanine phosphoribosyltransferase; UBC: ubiquitin C; U, untreated control

Since we found that PS-Eu are internalized by cells, we evaluated the response of quasi-ALI Calu-3 cultures to particle treatment. We assessed the induction of several antioxidant, pro- and anti-inflammatory biomarkers at the mRNA level with RT-qPCR after 24 h of exposure to increasing doses of PS-Eu (1.5–45 µg/cm²) and positive control staurosporine (0.75µM) (Fig. 13.A). These markers are important because they are involved in mucosal immunity and antioxidant defence in the human lungs. The tested markers were: interleukin (IL) 8 (IL-8) and 6 (IL-6), monocyte chemoattractant protein-1 (MCP-1), chemokine (C-C motif) ligand 5 (CCL5), tumour necrosis factor-α (TNF-α) and transforming growth factor-β (TGF-β). IL-8 and IL-6 protein release was also quantified via ELISA and results are included in Additional File 3. Exposure to PS-Eu at 1.5 and 5 µg/cm² significantly induced the expression of IL-6 mRNA, and TGF- β mRNA was also significantly increased at 5 µg/cm². However, TGF- β induction was low (< 2-fold change), and higher concentrations had no effect pointing to a possible hormesis response. Slight but not significant increases of IL-6 and IL-8 release were observed by ELISA, which followed the same trend found in RT-qPCR (Additional File 3). The positive control staurosporine increased significantly the mRNA expression of IL-8, IL-6, TNF-α and TGF-β (> 2-fold change for IL-8 and TNF-α) and reduced significantly the expression of CCL5. Oxidative stress, another cellular stress response that might be induced by nanoplastics, was assessed by analysing the expression of several antioxidant enzymes mRNAs at 24 h of exposure (Fig. 13.B). mRNA levels of superoxide dismutase 2 (SOD2), heme-oxygenase 1 (HO-1) and NAD(P)H quinone dehydrogenase 1 (NQO-1) remained, similarly to ROS production in A549 cells, stable at tested concentrations and time points, while staurosporine induced SOD2 mRNA.

**Fig. 14:**
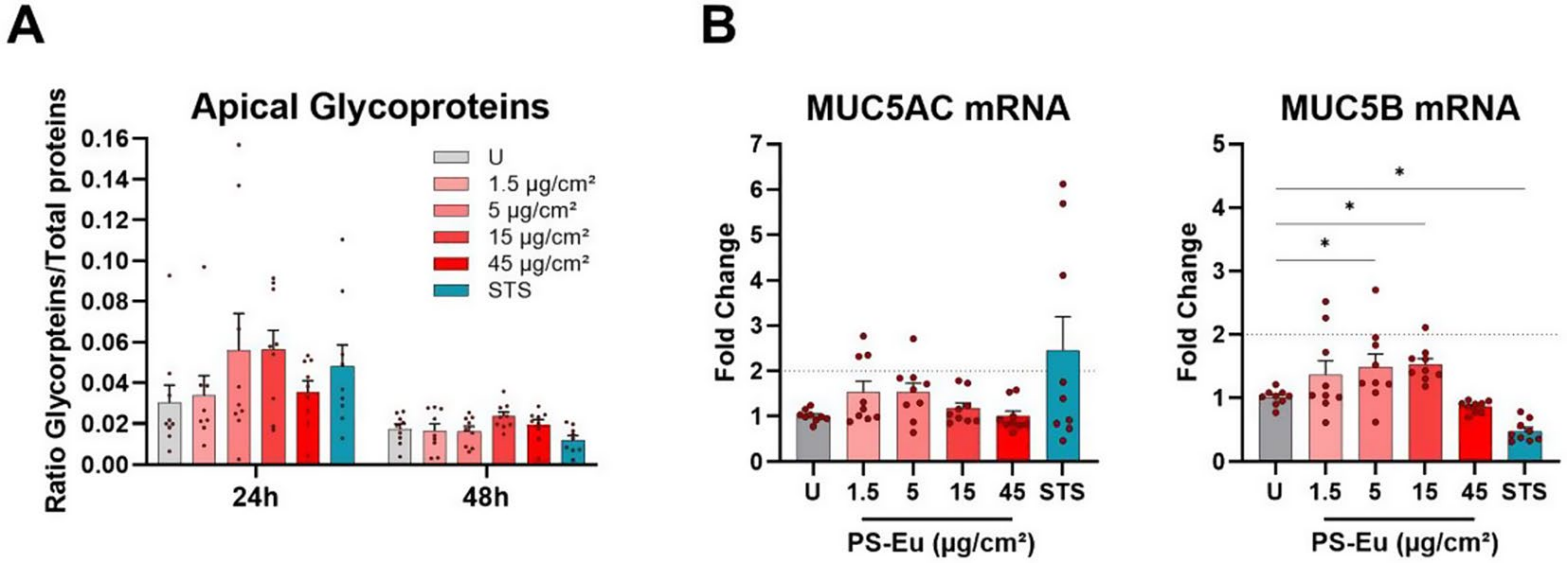
Glycoprotein production and mucin gene expression of quasi-ALI-grown Calu-3 cultures treated with PS-Eu. Calu-3 cells were cultured in quasi-air-liquid interface conditions (ALI) for 7 days before 24 h treatment with droplets (18 µl/cm^2^) of PS-Eu. Particles were applied from 1.5 to 45 µg/cm² and 0.75 µM staurosporine (STS) was used as positive control. After exposure, glycoprotein content of the apical secretome was quantified by ELLA **(A)** and reported to the total protein content of each transwell measured by bicinchoninic acid assay (BCA). RT-qPCR analysis was performed using UBC and HPRT as reference genes. Mucin mRNA expressions **(B)** is shown as fold change of control. 3 independent experiments with 3 technical replicates were performed and reported as mean ± standard error of mean. Statistical analysis was performed with one-way ANOVA. Abbreviations: Calu-3: cultured human airway epithelial cells; PS-Eu, europium doped polystyrene nanoparticles; ELLA: enzyme-linked lectin assay; HPRT: hypoxanthine guanine phosphoribosyl transferase; UBC: ubiquitin C; MUC: mucin; mRNA: messenger ribonucleic acid; U, untreated control

Finally, mucus secretion, a cell function-specific biomarker which is critically involved in epithelial barrier function, was analysed by quantifying glycoproteins in the apical secretome with enzyme-linked lectin assay (ELLA) at 24 h and 48 h of exposure (Fig. 14.A). A modest trend to hypersecretion of mucins reflected by the increase in glycoproteins was observed at 24 h of exposure to 5 and 15 µg/cm² and at 48 h of exposure to 15 µg/cm² and 45 µg/cm², that although was not statistically significant. To confirm this trend, we evaluated the induction of 2 mucins, mucin (MUC) 5AC and MUC5B mRNAs at 24 h of exposure (Fig. 14.B). Expression of both mucin mRNA had the same trend observed with ELLA assay, with statistically significant induction of MUC5B mRNA at 5 and 15 µg/cm² but induction was below 2-fold change.

## Discussion

In this study, we evaluated the effects of model polystyrene nanoparticles doped with europium on human lung cells, using alveolar (A549) and bronchial (Calu-3) cell models grown under both 2D and 3D conditions. The goal was to deepen our understanding of nanoplastic hazards and investigate potential adverse outcomes associated with exposure.

To do so, after in-dept physicochemical characterization of the particles, we first assessed their impact on cell viability and barrier integrity using LDH, Resazurin, NRU and Ly assays, along with TEER measurements and ROS quantification in particle-exposed cells and found no signs of cytotoxicity or barrier damage. Additionally, by performing a spectrofluorimetric analysis aiming to detect europium fluorescence in the basolateral side of transwells inserts, we could show that particles did not translocate across the epithelial layer, further corroborating the idea of an intact epithelial barrier in both models.

By using transmission electron microscopy (TEM) analysis however, we could detect a dose- and cell-dependent uptake of PS-Eu in both models and marked ultrastructural alterations especially in A549 cells cultured under 3D conditions. In these cells, we observed a loss of mitochondria-associated ER membranes (MAMs) and lamellar bodies (LBs) - structures involved in surfactant production - along with the appearance of glycogen-like aggregates. These changes were associated with a significant increase in both the number and size of digestive and acidic vesicles, which correlates with the enhanced staining incorporation observed in the neutral red uptake (NRU) assay. Remarkably, these vesicles were frequently found filled with PS-Eu and LBs undergoing digestion suggesting that PS-Eu may influence surfactant production, a cell-specific function in A549 cells.

Additionally, based on these results and the performance of the individual cytotoxicity assays (LDH, Resazurin, and NRU), we might suggest that NRU assay is the most sensitive method for detecting the earliest effects of particle-cell interactions. In fact, such process typically begins with particle contact and endocytosis, followed by their encapsulation in endosomes, which eventually mature into acidic lysosomes that can be detected by the NRU assay.

The ability of PS-Eu to affect surfactant production was confirmed by detailed quantification of surfactant proteins (SPs) by ELISA. This analysis demonstrated that the intracellular pool of surfactant proteins, particularly SP-B, was significantly affected by the treatment in transwells-grown A549 cells. However, the impact on the extracellular pool was minimal, especially in quasi-ALI cultures. This was, to our knowledge, the first time that such a broad number of SPs was tested in particle-treated cells, as up to now, other groups were mainly focusing on studying nanoplastic-SP interactions in acellular models [53].

Overall, quasi-ALI cultures, after being grown in more physiological settings, looked less susceptible to PS-Eu exposure compared to submerged cells, further supporting the use of relevant culture systems to test particle hazard on lung models. Interestingly, the phenotype observed in quasi-ALI cells, which was accompanied by glycogen-like aggregates, closely resembles that found in SP-B deficient mice [50], highlighting the crucial role of SP-B and its proprotein in lamellar body formation [14].

Our surfactant protein data, however, appeared to contrast with our surface tension (ST) results, where we observed reduced tension, which is usually a sign of increase surfactant secretion. This discrepancy could be explained by the possibility that PS-Eu, after interacting with the cell, may induce an early stress-induced release of LBs, leading to a decrease in surface tension before affecting the endosomal compartment after uptake. In line with this, we can suppose that at later time points, the changes detected in the intracellular pool (composed by recycled and newly synthetized surfactant proteins) will affect the extracellular one in the long term, ultimately hampering the ability of A549 to control surface tension.

Of interest in this context is the work of Siops et al. [54], which showed that after uptake, PS particles were able to induce autophagy and lysosome exocytosis in rat alveolar epithelial cells. However, in this paper, lysosome secretion was detected using the LysoTracker Green staining, which is known to also stain LBs [55, 56]. It therefore cannot be excluded that other acidic organelles, like LBs are being exocytosed in such conditions and that this can occur also in our setting causing a decrease of surface tension in the short term. In line with the work of Siops et al. [54], we also found the presence of secretory lysosomes in quasi-ALI-treated cells.

One well-known trigger for surfactant release is extracellular ATP [56, 57], which can also be secreted in response to mechanical stimuli, such as interactions with cigarette-smoke MPs/NPs [58]. Interestingly, ATP production appears to be upregulated in treated cells, as suggested by the increased metabolic/mitochondrial activity observed in the Resazurin assay, although the underlying cause of this increase remains unclear.

Mitochondria, being involved in ATP production, are often known to change the intensity of their activity to compensate new energetic requirements of the cell (e.g. surfactants production/recycling), or to counteract stressful conditions, such as particle uptake/degradation [59–61] which usually is achieved through the ATP-dependent process of lysosomal acidification [62]. Therefore, Resazurin conversion can serve both as viability biomarker (when decreased) and a stress-related biomarker (when increased).

However, since we do not observe an increases in the extracellular poll of surface-active surfactants (e.g. SP-B), but measure a lower surface tension, we might speculate that this process is SP-B independent and caused by a change in surfactant composition, especially in terms of surfactant lipids, which are secreted by LBs and make up a large part of the surfactant pool but were not specifically analysed in this study [63]. In fact, it is known that mammalian surfactants can have a wide range of phospholipid concentrations, which, together with surfactant proteins (e.g. SP-B) and surfactant reservoirs, play an important role in surface tension reduction [63]. In fact, increasing the amount of phospholipid in the monolayer will progressively lower the surface tension, with the degree of reduction varying based on phospholipid composition [64]. Therefore, it can be suggested that an altered lipid composition is decisive for the reduction of surface tension in this case. This process might be relevant, especially for the quasi-ALI counterpart where we observed a dose-dependent reduction of lamellar bodies as visualized by TEM. This already indicates that there are also differences in the phospholipid composition that could have influenced the function of the surfactant.

In this context, since SP-B is the protein mostly influenced by particle treatment in quasi-ALI and submerged 3D samples we can hypothesize that SP-C [65], another surface-active SP, which has a superior role in the formation of the surfactant reservoir and reinsertion of lipids [64], is replacing SP-B with lower efficiency in the tension lowering process [66–68]. However, due to the limited amount of material available to be analysed, we were not able to systematically analyse this aspect.

For what concerns SP-A instead, it is know that this protein in combination with SP-B causes a selective adsorption of surface-active lipids (Dipalmitoylphosphatidylcholine, DPPC) into the surface film [69]. However, since SP-B concentration was generally reduced in our experiments it can be assumed that SP-A has no surface-active effect in such conditions, but it rather induces an immunological response to the particles. The intracellular reduction of SP-B might explain the marked reduction of lamellar bodies in treated quasi-ALI samples [50, 69].

SP-G and SP-H are two not yet well characterized surfactant proteins that are surface active and have an immunoregulatory role [70, 71]. In our experiments, there were slightly significant reductions in the intracellular pool and the complete loss of SP-G in the supernatant, which might have also influenced surface tension and immunological activity.

These results highlight our lack of understanding of how surfactants stabilize surface tension in healthy lungs and how this stability is disrupted by exogenous influence. Thus, further experiments must follow to explain the effects. However, we can conclude that SP production could be a sensitive and important cell-specific function biomarker for assessing the effects of nanoplastics on alveolar lung models.

When we assessed the impact of PS-Eu on Calu-3 cells instead, TEM examination showed that particles are mainly internalised as single particles by the apical cells and then vesicles fused into larger endosomes in the perinuclear area which may also present glycogen granules. Interestingly, PS-Eu were not found in mucus vesicles which, in contrast to surfactant, are not recycled from the extracellular lining fluid. PS-Eu uptake in quasi-ALI culture seem higher at earlier timepoints when compared to later ones, suggesting either cell death or particle exocytosis. This is corroborated by TEM images showing that some extracellular particles were covered with granules resembling glycogen. These results are in line with the work of Paget and Dekali, which showed how Calu-3 after an initial particle uptake tend to release the particles [72].

Upon interaction with cells, PS-Eu are able to increase the expression of MUC5B, ZO-1, IL-6 and TGF-β mRNA, but this induction was not observed at higher concentrations and, except for IL-6, was below 2-fold change over the control. The increase of apical glycoprotein was not statistically significant which could be due to the lack of induction of other mucins like MUC5AC mRNA whose expression was unchanged by PS-Eu exposure. Our results suggest that PS-Eu can initiate a moderate pro-inflammatory response which cannot be linked to microbiota contamination, as there was no TLR activation by the NPs. Interestingly IL-6 which is known to increase MUC5B production in an autocrine/paracrine manner [73] is among the most increased cytokines in chronically exposed employees at the workplace [74]. Thus, in future, it would be relevant to assess the impact of PS NPs on alveolar and bronchial models under chronic exposure conditions. Alveolar cells could then be exposed to low particle concentrations that may accumulate over time, more accurately reflecting real-life scenarios. However, in this study, we used high particle concentrations but in a broad range to assess acute dose-response relationships, as our primary objective was to investigate the mechanistic effects of PS NP-cell interactions. The concentrations used ranged from 0.48 µg/cm² in submerged 2D A549 cultures and 1.5 µg/cm² for Calu-3 ALI cultures - considered relatively low for acute in vitro experiments (see dose conversion in Table 1) - to 45–75 µg/cm² in quasi-ALI cultures of Calu-3 and A549 cells, respectively. Interestingly, in some cases, lower concentrations within this range were more effective than higher ones, as demonstrated by RT-qPCR results.

## Conclusion

### Main findings

In this study, we demonstrated, in line with previous findings, that carboxylated polystyrene nanoparticles do not induce acute cytotoxicity or barrier damage across a broad concentration range in alveolar (A549) and bronchial (Calu-3) cell models, grown under both quasi-ALI and submerged conditions [49]. However, we showed here that PS-Eu are able to target cell-specific functions such as surfactant, mucus and cytokine production supporting the idea that standard cytotoxicity tests are not sufficient to inform particle hazard and that additional groups of biomarkers should be investigated. These biomarkers are listed and described in the following section.

### Classes of biomarkers for identification of effects and informing hazard

**Fig. 15:**
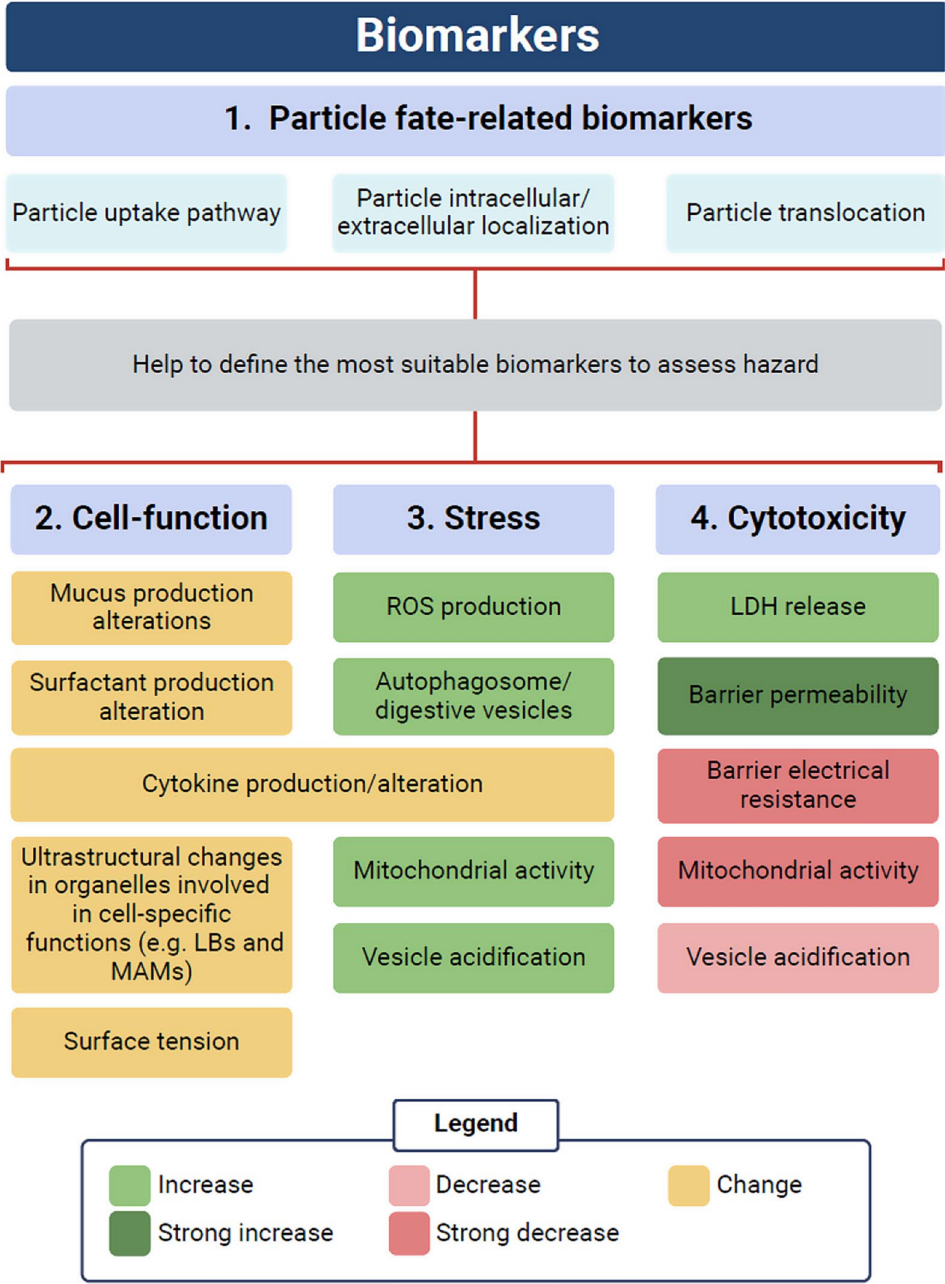
graphical depiction of the proposed classes of biomarkers

When assessing the hazard of micro- or nanoplastics in vitro, it is important to remember that most frequently only cytotoxicity is assessed, however even minor changes in cell functionality could have long-term effects on lung activity. Although considered biocompatible, polystyrene nanoparticles are not readily biodegradable [2, 4], and are therefore likely to persist in the lung or associated immune cells and tissues for prolonged periods of times. Thus, in the paper presented here, we defined different classes of biomarkers to assess hazard when using lung in vitro models. Some biomarkers may belong to more than one category (Fig. 15).

These categories are:

- **Cell-specific function biomarkers** such as: surfactant or mucus production, surface tension and cytokine production. These biomarkers are model-specific and target the main function of a given cell type. Alveolar epithelial cells, for example, play a critical role in surface tension control, and thus, investigating surfactant production, composition and secretion is critical to assess alterations of their activity.
- **Stress-response biomarkers** such as: ROS production, increased metabolic activity, increased number or size of acidic organelles (NRU uptake) and autophagosomes and presence of dilated ER cisternae and cytokine production. These changes can be detected in cells that try to counteract stressful conditions. For example, the excessive increase in metabolic activity or the number of acidic organelles can be a stress response to particle digestion. Similarly, autophagy is often initiated in the presence of low nutrient intake or to eliminate damaged organelles. Cellular stress response-biomarker are however not necessarily linked to cell death, as cells can recover and return to the baseline condition typical of unexposed cells. If the cell cannot repair the damage, cytotoxicity-related changes could be detected.
- **Cytotoxicity-related biomarkers** such as: LDH release (necrosis), chromatin condensation, significant loss of barrier integrity (as indicated by increased epithelial permeability and reduced trans-epithelial resistance) and loss of metabolic activity and/or NRU uptake (indicative of malfunctioning of mitochondria and the ATP-dependent proton pump) normalized by cell number or protein content. They are usually linked to cell death or significant epithelial barrier damage.
- **Particle fate-related biomarkers** such as: particle translocation, intracellular trafficking and localization, cell-specific uptake, uptake mechanisms, persistence in cells etc. These biomarkers can help in defining the pathways that might be affected by the treatment. For example, in the present publication, particles were found enclosed in digestive vesicles which contained also lamellar bodies. Thus, it was important to see if NRU uptake and surfactant production were affected.

We suggest that at least one biomarker per category should be addressed to exclude hazard, with a particular focus on those related to particle fate and cell-specific functions. Applying these rules, we could show here that PS-Eu do not affect cytotoxicity-related biomarkers but triggers a stress response in A549 cells that ultimately impair cell-specific functions up to a concentration of 100 µg/ml over 48 h of exposure. Finally, based on our results, we can conclude that the assessment of particle fate-related biomarkers is critical to better define the metabolic pathways potentially affected by particle interaction and to determine further experimental steps in the assessment of particle hazard.

## Materials and methods

### Nanoplastic characterization

Polystyrene-Europium (PS-Eu) Thermo Scientific™ Fluoro-Max™ Fluorescent Carboxylate-Modified Particles were purchased from Thermo Scientific. Subsequently they were characterized using different methods. This test material was used in collaborative work between different labs within the PlasticsFatE project (Grant Agreement n° 965367). Carboxylated PS-Eu nanoparticles were chosen as model particles in this study due to their ability to be synthesized as a highly monodisperse population, ensuring a uniform particle sample. The carboxylated surface enhances stability under culture conditions, minimizing aggregation and enabling researchers to investigate particle-induced effects without the confounding influence of aggregation-related artifacts. Furthermore, it has been demonstrated that during aging, PS particles can naturally form carboxyl groups on their surface [75]. 

#### Electron microscopy of particles via scanning electron microscopy (SEM)

5 µl of PS-Eu stock suspension were pipetted on a support and let dry overnight in a desiccator. The samples were coated with gold-palladium using the Gatan Precision Etching Coating System (PECS) 682 (USA), then they were imaged using a JSM-6500 F (JEOL) operated at 15 kV.

#### Size distribution Estimation via transmission electron microscopy (SEM)

To estimate the mean size distribution of PS-Eu particles, the diameter of 100 particles from different regions of the SEM grid were measured by ImageJ software.

#### Size distribution Estimation via nanoparticle tracking analysis (NTA)

Particle concentration and size distribution analysis in dispersion were performed by the ZetaView^®^ PMX-120 (Particle Metrix GmbH, Germany) nanoparticle tracking analyser, equipped with a light source set to a wavelength of 488 nm. Before the measurements, the samples were diluted in water: stock PS-Eu nanoplastics (10 mg/ml) 50000 times, while PS-Eu exposed to complete culture medium (CCM) (final polymer concentration 100 µg/ml) and CCM alone were diluted 500 times. After optimization of the instrumental parameters, the sensitivity and the shutter, were set at 70 and 100, respectively, in 33 videos of 1 s recorded in 11 different positions, recording a total number of particles of around 2000 per single measurement, each measurement was performed in triplicate. The signal of CCM alone was used as blank and subtracted from the size distribution of PS-Eu exposed to CCM. For the dosimetry measurements PS-Eu NPs were incubated with CCM at 37 °C and kept steady. Sampling (1% of the volume) of the upper part of the dispersion was performed at 0 and after 18.5 h and 42.5 h. The sampled dispersions were diluted 500 times in water and measured by NTA.

#### Dynamic light scattering (DLS)

Size distribution of PS-Eu stock solution was measured by dynamic light scattering (DLS) at 37 °C using micro Vis cuvettes (Eppendorf, Hambourg, Germany) with a Zetasizer Nano-ZS (Malvern Instruments, Ltd, Worcestershire, UK). PS-Eu NPs were diluted to 0.1 g/L in 100% milliQ H_2_O. Measurements were based on the Smoluchowski model.

#### Electrophoretic light scattering (ELS) and Z-potential estimation

Z potentials on NPs were measured using a Zetasizer, Nano ZS electrophoretic light scattering analyser (Malvern Instruments, Malvern, UK) equipped with a light source wavelength of 632.8 nm and a fixed scattering angle of 173^◦^. The samples were measured in triplicate at 25 °C. PS-Eu particles incubated with CCM and M were purified by centrifugation and resuspended in water before been measured. All measurement were performed at pH 7.4 and with similar conductivity: 1.3 ± 0.2 mS/cm.

#### Dosimetry in culture medium using the ISDD model

The deposited dose in culture medium condition was calculated either theoretically or experimentally. Briefly, we first calculated the theoretical deposited mass using the in vitro sedimentation, diffusion and dosimetry model (ISDD) [32] assuming a concentration of 100 µg/ml, a diameter of 279 nm (values obtained with NTA, Fig. 1.C), and a density of 1.05 g/cm^3^, which is the density of polystyrene. Simulation time was set at 48 h (48 points); dish dept 0.00833 m; volume 0.25 ml; Temperature 310 K. The data obtained provided the theoretical deposited mass of PS-Eu particles in CCM without a protein corona and was equal to 2,93 µg over 48 h (blue line, Fig. 1.E). Then we modelled the deposited mass of PS-Eu particles assuming a core-shell structure of 316 nm composed by a polystyrene core of 279 nm, and a compact protein corona shell of 18.5 nm (values obtained with NTA, Fig. 1.C.) characterized by a density of 1.37 g/cm^3^ (the average density of proteins [76]). The final assumed density of the object was 1.15 g/cm^3^. The data obtained estimated a deposited mass in CCM of 6,26 µg over 48 h (red line, Fig. 1.E). However, often the corona is not compact, and consequently might have a density which is lower than 1.37 g/cm^3^. Thus, we measured experimentally with NTA the deposited number of PS-Eu particles at 0, 18 and 42,5 h - than converted to mass - and found a quite linear correlation between the time and deposited mass, with 4.5 µg deposited over 42,5 h equal to a deposited dose of ~ 5 µg at 48 h. Interestingly, the deposited mass measured experimentally lays perfectly between the deposited mass calculated theoretically with ISSD for PS-Eu Stock (2,93 µg) and PS-Eu CCM (6,26 µg), suggesting that a protein corona is formed, but that it is not fully compact as we assumed for PS-Eu CCM. In the experiments performed, cell treatment with particles is indicated in µg/ml, however in Table 1 we provide a dose table to allow an easy conversion of µg/ml in different formats.

**Table 1:** Particle dose conversion table

Estimated dose							
**A549** **2D** **Submerged**	**Ps-Eu mass per volume (µg/ml)**	3.125	6.25	12.5	25	50	100
	**Mass applied (µg)**	0.4687	0.937	1.875	3.75	7.5	15
	**PS-Eu mass per area (µg/cm** ^**2**^ **)**	1.46	2.92	5.84	11.68	23.36	46.87
	**Approximation of (µg/cm** ^**2**^ **)**	1.5	3	5.5	11.5	23.5	47
**A549** **3D Submerged**	**Ps-Eu mass per volume (µg/ml)**	3.125	6.25	12.5	25	50	100
	**PS-Eu mass per area (µg/cm** ^**2**^ **)**	2.441	4.882	9.765	19.53	39.0625	78.125
	**Mass applied (µg)**	0.78	1.56	3.125	6.25	12.5	25
**A549** **3D** **quasi-ALI**	**PS-Eu mass per area (µg/cm** ^**2**^ **)**	2.441	4.882	9.765	19.53	39.0625	78.125
	**Mass applied (µg)**	0.78	1.56	3.125	6.25	12.5	25
**Calu-3** **3D** **quasi-ALI**	**PS-Eu mass per area (µg/cm** ^**2**^ **)**	1.5	5	15	45		
	**Mass applied (µg)**	1.68	5.6	16.8	50.4		
**Delivered dose – (measured experimentally in submerged conditions combining ISDD with NTA)**							
**A549** **2D** **Submerged**	**PS mass deposited/well in 48 h (µg)**	0.156	0.3125	0.625	1.25	2.5	5
	**PS mass deposited/area in 48 h (µg/cm** ^**2**^ **)**	0.487	0.975	1.95	3.9	7.8	15.6
**A549** **3D Submerged**	**PS mass deposited/well in 48 h (µg)**	0.156	0.3125	0.625	1.25	2.5	5
	**PS mass deposited/area in 48 h (µg/cm** ^**2**^ **)**	0.487	0.975	1.95	3.9	7.8	15.6
**A549** **3D** **quasi-ALI**	**PS mass deposited/well in 48 h (µg)**	0.78	1.56	3.125	6.25	12.5	25
	**PS mass deposited/area in 48 h (µg/cm** ^**2**^ **)**	2.441	4.882	9.765	19.53	39.0625	78.125
**Calu-3** **3D** **quasi-ALI**	**PS mass deposited/well in 48 h (µg)**	1.68	5,6	16.8	50.4		
	**PS mass deposited/area in 48 h (µg/cm** ^**2**^ **)**	1.5	5	15	45		

Above dose conversion table is shown. Doses are presented both in terms of estimated and delivered dose. The delivered dose is calculated experimentally in submerged samples using the ISDD model and NTA. In quasi-ALI samples instead, due to the low volume of liquid in the apical compartment, it is assumed that all the particles applied will meet cells and thus be delivered. Doses are shown in terms of (1) Estimated doses - applied mass (µg), mass per volume (µg/ml), mass per area (µg/cm^2^); (2) Delivered doses - mass deposited/well (µg) in 48 h (assuming a well area of 0,32 cm^2^), mass deposited/area (µg/cm^2^) in 48 h. Note: In submerged samples, if the same concentration is applied (µg/ml) and wells with the sample surface area are used the amount of delivered particles is the same between 3D-grown samples (grown using transwells) and standard 2D submerged samples

#### TLR2 and TLR4 activation assay

This assay is based on human embryonic kidney (HEK293) reporter cells which upon exposure and activation of Toll-like receptor 2 (TLR2) and 4 (TLR4) by their respective microbial ligands (lipoteichoic acid, LTA and lipopolysaccharide, LPS), release the secreted embryonic alkaline phosphatase (SEAP) that produces a colorimetric reaction thanks to the presence of its substrate in the cell culture media. For the present study, exposure experiments with parental HEK293 cells, TLR2 and TLR4 reporter HEK293 cells (Invivogen, France) were conducted in a 96-well plate following the procedure previously reported by Afanou et al. 2023 [77]. Briefly, 50.400 cells/well resuspended in 180 µl enriched cell medium (DMEM + 10% fetal bovine serum + mandatory HEK Blue selection antibiotics) were exposed to 20 µl of the PS-Eu particles suspensions at doses ranging between 0 and 200 µg/ml (equal to 0 -1.33 × 10^9^ particle/ml). The cells were then incubated at 37 °C, 5% CO_2_ and high relative humidity conditions for 24 h. Following the incubation, 20 µl of the cell supernatant were carefully transferred to new microplates and completed with 180 µl Quanti-Blue detection reagent (Invivogen, France). After 180 min incubation, the colour was spectrophotometrically measured at 649 nm using Synergy Neo 2 multimode microplate reader with Gen 5 data analysis software (BioTek Instruments GmbH, Bad Friedrichshall, Germany). Each sample was run in triplicate, and the whole experiment was repeated three times. The data are reported as mean fold change related to the parental cells (similar to the negative controls). Negative controls (Endotoxin free water) and positive controls for TLR2 (ultrapure LTA, lipoteichoic acid; InvivoGen, France) and TLR4 (ultrapure LPS, lipopolysaccharide; InvivoGen, France) activation were included in all experiments.

#### PS-Eu fluorescence emission and excitation spectra acquisition

100 µg/ml PS-Eu fluorescence emission and excitation spectra were collected using a Cytation 3 (Invivogen) after 48 h incubation in complete culture medium (CCM) at 5% CO_2_, 37 °C and humified conditions.

#### Transmission Electron microscopy Energy-Dispersive X-Ray spectroscopy (TEM-EDX)

For particle characterization, washed PS-Eu NPs were diluted in milliQ H_2_O, deposited on a Cu-C Formvar grid. 2% uranyl acetate was added, and NPs were air dried then observed with TEM EDX detector. PS-Eu NPs EDX spectra were acquired using an Xplore detector (Oxford Instruments).

### Cell culture and exposure to particles

#### A549 cell culture and exposure to particles in 2D submerged conditions

100 µl of A549 cells in (ATCC) complete culture medium (DMEM, high glucose (Sigma), 4 mM L-glutamine (Sigma), 10% v/v FBS (Sigma)) were seeded at a concentration of 90,000 cell/ml in 96-well plates (TPP) and grown 24 h at 37 °C, and 5% CO_2_ in humified conditions. In parallel background controls wells containing exclusively complete medium were prepared. One day after seeding cells and medium wells were either left untreated or incubated with increasing concentrations of PS-Eu particles diluted in complete culture medium at final concentrations ranging from 3,125 to 100 µg/ml. An H_2_O_2_ positive control was also prepared by exposing cells to a final concentration of hydrogen peroxide of 0.05 mM. After 48 h Triton X-100 0,1% was added to half of the untreated wells and the plate was incubated for 30 min at 37 °C, and 5% CO_2_ in humified conditions. Cells response to particles was investigated by performing sequentially on the same plate: lactate dehydrogenase (LDH) assay, Resazurin assay, Neutral Red uptake (NRU) assay and Coomassie brilliant blue (CBB) assay. Alternatively, ROS production and particle uptake were estimated via flow cytometry after 30 min of treatment on the same type of cells (see Additional File 2).

#### A549 cell culture and exposure to particles in 24- and 12-well plate transwells

A549 cells in CCM were seeded at a concentration of 70,000 cell/ml in the top (apical) compartment of transwells (Sarstedt, PET, 3 μm pore size) and then grown for 10 days in submerged conditions in CCM (24-well plate transwells: top compartment: 250 µl, bottom (basolateral): 1000 µl. 12-well plate transwells: top 900 µl, bottom 1800 µl). The medium was replaced on day 3, 7 and 10 after seeding. On the same days, in 24-well plates grown cells, TEER, epithelial permeability, or tight junction expression were measured to monitor epithelial barrier formation over time for model characterization (see Additional File 2).

Starting from day 10 post seeding, samples were grown for an additional 7 days either in air-liquid interface (ALI) conditions (24 well-plate: top empty, bottom 1000 µl. 12-well plate: top empty, bottom:1800 µl) or submerged ones (24 well-plate: top 250 µl, bottom 1000 µl. 12-well plate: top 900 µl, bottom 1800 µl) and was changed every alternate day. In parallel to the stated samples, extra wells were prepared to serve as a control for future experiments: cell-free wells, and cell-containing wells.

On day 17, after measuring TEER and epithelial permeability, cells were exposed to plastic particles in duplicates. First, medium was changed in all bottom compartments. Then in the top compartment cells were either left untreated (medium change only) or incubated with particles at different concentrations (100 µg/ml, 50 µg/ml, 25 µg/ml) for 48 h in humified conditions at 37 °C, and 5% CO_2_. In submerged samples, particles were diluted in 250 µl (24-well-plate) or 900 µl (12-well plate) of CCM while in ALI-grown cells, cells were treated either with 20 µl (24-well plate) or 80 µl (12-well plate) of sterile-filtered milliQ water, the particle solvent (untreated control), or with water containing the same number of particles used for the submerged samples. This treatment is defined as quasi air-liquid interface (ALI). Medium was replaced also in control wells. After 48 h treatment, the response of cells to particles was investigated.

In 24-well plates first half of untreated control wells are incubated with Triton X-100 (0.1% v/v, Roche) for 30 min and then different assays were performed sequentially: LDH assay, lucifer yellow assay, TEER measurement and Resazurin assay. In 12-well plates instead cell ultrastructure (TEM), surface tension, particle translocation and surfactant protein production were investigated.

#### Preparation of PS-Eu for treatment of Calu-3

Initial PS-Eu NPs were washed twice by dilution at 1/5 in autoclaved miliQ water and centrifugation for 5 min at 15000 g at 4 °C to remove sodium azide from the dispersant (deionized water, 0.05% azide). The PS-Eu NPs pellet was then resuspended in autoclaved milliQ H_2_O. The final concentration of washed PS-Eu NPs was determined by DLS using the initial commercial suspension to establish a calibration curve after verification of similar HD.

#### Calu-3 2D and 3D cultures and exposure to particles

The Calu-3 human adenocarcinoma epithelial cell line (ATCC HTB-55) was used from passage 24 to 28 according to Sanchez-Guzman et al. 2021 [78]. Calu-3 cells were cultured in Eagle’s Minimum Essential Medium (MEM, Gibco, Thermo-Fisher, France) supplemented with 10% v/v foetal bovine serum (FBS) (F7524, Merck, France), 1% nn-essential amino acids (NEAA) 100×, 1% sdium pyruvate, 1% Gutamax, 1% pnicillin streptomycin 100 ×, and 1% HPES buffer 100 × (all from Gibco, Thermo-Fisher, France). Cells were cultured at a density of 40000 cells/cm² in 25–75 cm² culture flasks (Costar, Corning, France) at 37 °C in a humidified 5% CO_2_ atmosphere and passaged weekly before confluence. 2D cultures were done on round glass slides placed in a 24-wells plate (Costar) at a density of 200000 cells/cm² in 250 µl of MEM supplemented with 10% FBS. Two days later cells were exposed for 24 h to PS-Eu NPs diluted in MEM supplemented with 4% FBS (200 µl per well) and fixed for TEM. To reconstruct the epithelial barrier, Calu-3 cells were seeded on transwells polycarbonate inserts in 12-well plate with a 3 μm pores diameter (Costar) and a 1.12 cm² surface area. Before seeding, transwells inserts were hydrated with 1.5 ml of Hank’s balanced salt solution containing Ca and Mg (HBSS ^Ca2+/Mg2+^, Gibco, Thermo-Fisher) in the lower compartment and 0.5 ml in the upper compartment for 15–30 min at room temperature. Cells are then seeded at a density of 50000 cells/transwell (apical: 0.5 ml of cell suspension, basolateral: 1.5 ml of MEM supplemented with 10% FBS), with medium change every 2–3 days. After 14 days, the apical medium was removed, and cells were cultured at the air-liquid interface for 1 week. The basolateral compartment was replaced by MEM supplemented with 4% FBS one day after creation of the air-liquid interface. At day 20, barrier permeability (lucifer yellow assay) and metabolic activity (AlamarBlue™ assay) were measured, and cells were exposed the next day. Exposures were done by adding droplets of PS-Eu suspensions diluted in HBSS ^Ca2+/Mg2+^ at 70% in water on the apical compartment (18 µl/cm²) or by adding the positive controls staurosporine (STS, S4400 Merck, stock solution at 2mM in DMSO) at 0.75 µM or Triton X-100 (Tx, Euromedex, Souffelweyersheim France) at 0.01% t the culture media of the basal compartment. After 24–48 h, the apical secretome was collected by rinsing with 200 µl HBSS ^Ca2+/Mg2+^, the basolateral media was collected, and both compartments were stored at -20 °C for further analysis. Subsequently, AlamarBlue and Ly assay were performed, and cells were then either lysed for mRNA quantification by RT-qPCR or lysed with milliQ H_2_O (100 µl) to quantify intracellular PS-Eu or fixed for TEM and Raman microscopy analysis. For more information see Additional File 4.

### Assessment of cell response to particle treatment

#### Lactate dehydrogenase (LDH) assay on 2D-grown A549 cells

Cell necrosis was assessed via the quantification of lactate dehydrogenase (LDH) release using the Cytotoxicity Detection KitPLUS (Roche). Briefly, after 48 h of treatment, half of the untreated cells were lysed with 0.1% (v/v) Triton X-100. Afterward, 50 µl of supernatants from each well were transferred to a fresh plate and incubated with 50 µl of the reaction mix provided in the kit for 30 min, at RT, light protected. Then, 25 µl of stop solution were added to each well, and absorbance was measured at 490 nm (Tecan Infinite F Nano+). Triton-treated wells served as control for maximum LDH release while cell-free wells incubated with or without particles served as background control for the correspondent treatments.

#### Resazurin, neutral red uptake, and coomassie brilliant blue assays on 2D-grown A549 cells

After using part of the supernatant for the LDH assay, all wells were refilled 50 µl of fresh CCM and mitochondrial and acidic organelle activity was measured via Resazurin and Neutral Red uptake assays. Briefly, a mix of resazurin (0.15 µg/ml in sterile PBS, Sigma) and Neutral red (0.4 µg/ml in sterile PBS, filtered at 0.2 μm from Sigma) in a ratio 3:2 was prepared and 50 µl of it were added to each well. Samples were subsequently incubated under humidified conditions at 37 ºC and 5% CO_2_ for 4 h and then cell metabolic activity was measured at Ex: 560/ Em: 590 nm (Tecan Infinite F Nano+). The liquid present in each well was then aspirated and 60 µl of Neutral red (NR) solvent (50% EtOH, 49% dH_2_0, and 1% acetic acid (Sigma-Aldrich)) were added to each well and samples incubated for 20 min at RT. Acidic organelle activity was then quantified using the same spectrophotometer set at Ex: 530/Em: 645 nm. Afterwards, protein content was estimated via Coomassie brilliant blue assay. Again, the supernatant was aspirated and 60 µl of Coomassie brilliant blue 6250 (CBB) staining (0.05% Coomassie blue 6250 (Merc) in 30% methanol (Honeywell), 10% acetic acid and 60% milliQ water, 0.2 μm filtered) was added to each well and samples were incubated for 30 min at RT. After eliminating the supernatant, 60 µl of CBB solvent (0.1 M NaOH (Signa-Aldrich) in dH_2_0) were added to each well for 20 min. Then 60 µl of NR solvent were added on the top of all samples and absorbance was measured at 595 nm after carefully shaking the plate. In all assays Triton-treated wells served as control for minimum NRU, CBB or Resazurin signal while cell-free wells incubated with or without particles served as background control for the respective treatments.

#### ROS quantification and particle uptake estimation via flow cytometry on 2D-grown A549 cells

ROS production and particle uptake estimation were performed via flow cytometry using a BD FACS Melody. Briefly, A549 were first detached using Triple Select (Gibco) and then 32 × 10^4^ cells were transferred in FACS tubes to be washed once with PBS (Sigma). Then samples were resuspended in a 20 mM solution of the general ROS indicator CM-H_2_DCFDA (Invitrogen Thermo Fisher), and incubated for 30 min at 37 °C, 5% CO_2_ in humified conditions. Once stained, samples were washed once more with PBS to be then either left untreated or incubated with increasing concentrations of PS-Eu particles (from 3,125 to 100 µg/ml) in CCM for additional 30 min at 37 °C, 5% CO_2_, humified conditions. 0.05 mM H_2_O_2_-treated cells served as control for ROS production. Finally, ROS production was quantified via flow cytometry after doublets exclusion and particle uptake was indirectly estimated by collecting side scatter values (SSC).

#### Preparation of transwells-grown A549 cells for confocal microscopy

The staining protocol used in this manuscript is a modification of the one published by Buckley et al. 2018 [79]. Briefly, sample fixation was performed for 10 min at − 20 °C directly on the 24-well plate plastic transwells using ice-cold 100% mthanol (Honeywell): acetone (Merk) 1:1, followed by 6 washing steps with PBS (Sigma) at RT (5 min each). To minimize non-specific binding of antibodies, a blocking solution containing 20% FBS (Sigma) and 1% BSA (Sigma) in PBS was added to cells for 30 min at RT. Samples were then incubated with anti-ZO-1 5 μg/ml Alexa Fluor 488 (Invitrogen, clone ZO1-1A12) and anti-Occludin 5 μg/ml Alexa Fluor 594 (Invitrogen, clone OC-3F10) in PBS for 1 h at RT, and washed with PBS for 50 min at RT. Again, during this period the buffer was replaced every 10 min. Once the washing step was completed, cell nuclei were stained with DAPI (1 µg/ml) in PBS for 10 min at RT. After discarding the liquid, membranes were cut from the transwells using a scalpel and mounted onto glass slides with natural mounting medium (Sigma). Confocal laser scanning microscopy (CLSM) was performed with an Axio Observer Z1 inverted microscope equipped with LSM 800 (ZEISS), using an immersion 100x magnification objective. The Alexa Fluor 488 signal was excited with a diode laser at a wavelength of 488 nm (green laser) with 3% intensity, Alexa Fluor 594 signal was excited with a diode laser at a wavelength of 561 nm (red laser) with 5% intensity, and the DAPI signal was excited with a diode laser at a wavelength of 405 nm (blue laser) with 3% intensity, and a pinhole aperture of 35 μm. At least three sets of images (512 × 512 pixels) were acquired per membrane. When over time confocal images were collected, cells were seeded at different time points and cultured for a given amount of days (e.g. 1, 3, 7, and 10) and then harvested, stained and imaged on the same day using this protocol.

#### Preparation of transwells-grown A549 cells for scanning electron microscopy (SEM)

The morphology of A549 cells grown for 17 days on 12-well plate transwells was investigated via SEM. Cells were immersed in Karnovski fixative (25% glutaraldehyde, 8% paraformaldehyde in Na-P buffer) and incubated overnight at 4 °C after the elimination of the cell medium. The day after, samples were washed 3 times with Na-P buffer (10 min each) before being incubated for 1 h with 1% OsO_4_ in dH_2_O. Osmium impregnation was then followed by 3 additional washing steps with dH_2_O before samples were dehydrated using ethanol series (10 min each, with 30%, 50% 70% and 90% EtOH). Finally, after two washes with absolute ethanol (10 min each) (Honeywell), transwells were incubated for 10 min with increasing concentrations of HDMS in ethanol (EtOH: HMDS ratios 7:3, 1:1 and pure HDMS). Lastly, pure HDMS was added to each well and let air dry overnight. Once ready, samples were detached from the transwells using a scalpel and mounted on supports. Samples were covered with a 5 nm gold/palladium layer using a Gatan PECS 682 operated at at 10 kV in argon atmosphere before they were imaged using a JSM-6500 F (JEOL) operated at 5 kV.

#### Lactate dehydrogenase (LDH) assay on transwells-grown A549 cells

Since LDH was measured in 24-well plate transwells, 250 µL of fresh medium were added to the apical compartment of quasi-ALI wells to equalize medium volumes with the submerged samples. Then, Triton X-100 (0.1% v/v, Roche) was added to half of untreated control cells. After 30 min of incubation at RT, supernatants from the apical and basolateral compartments were mixed and transferred into a fresh 96-well plate (TPP) (50 µL each, four replicates), where they were tested for LDH release as described in Sect. 5.3.1. This was done because LDH can be released, upon cell death, both in the apical and basolateral side through the membrane. Triton-treated samples served as control for maximum LDH release. LDH measurement was then followed in by lucifer yellow assay.

#### Lucifer yellow (Ly) assay performed on transwells-grown A549 and Calu-3 cells

In transwells-grown A549 cells, epithelial permeability was measured via lucifer yellow (Ly) assay either during cell culture (from day 1 to 17 after seeding) or upon 48 h treatment with particles, after performing LDH assay. (NOTE: if permeability was measured to monitor barrier formation over time during cell culture, the Triton X-100 incubation step mentioned in the previous section was not performed. This was done only after 48 h of incubation with the test sample). After apical and basolateral supernatants were mixed and used for LDH assay, transwells were washed twice with HBSS^Ca2+ Mg2+^ (Gibco) and transferred to a fresh 24-well plate. Here 1000 µL of HBSS were added in the basolateral compartment while 200 µL of sterile-filtered Ly (0.1 mg/ml, Merk) in HBSS were added on the apical compartment. Samples were then incubated for 1 h at 37 °C, 5% CO_2_, in humified conditions. Subsequently, 150 µL of supernatant from the basolateral side were transferred in duplicates to a fresh 96-well plate. Fluorescence signal was acquired using a Tecan Infinite F Nano + plate reader (Ex: 485; Em: 538) with the gain set on optimal. Medium transwells served as controls for 100% permeability. After measurement cells were washed twice with HBSS and moved back to their original 24-well plate. Medium was refreshed and TEER measurement was performed.

In Calu-3 cells instead, which were grown on 12-well plates transwells, 500 µl of Ly at 0.1 mg/ml in HBSS ^Ca2+/Mg2+^ were added to the apical compartment and 1 ml to the basolateral one. After 1 h incubation, 100 µl of the apical and basal solutions were transferred to a 96-well clear bottom microplate (µClear^®^) for fluorescence measurement on a FlexStation 3 multi-mode microplate reader (Ex: 485; Em: 538) and permeability was calculated according to (Sanchez-Guzman et al. 2021) [78].

#### Transepithelial electrical resistance (TEER) measurement on transwells-grown A549 cells

TEER was measured after performing Ly assay, using the Epithelial Volt/Ohm Meter 3 (EVOM3) (World Precision Instruments) following manufacturer’s instructions. EVOM3 was calibrated before each experiment, and cell-free transwells served as background control for TEER. Similarly, Triton X-100-treated samples served as a control for minimum electrical resistance. Untreated cells instead serve as control for maximum resistance. During data acquisition, the instrument was set on AUTO mode (resistance), and each well was measured twice. NOTE: prior measurement, when medium was changed in all samples after performing Ly assay, 250 µL of CCM were added in quasi-ALI cultures on the apical compartment to have a volume comparable to submerged samples. After measurement, when over time permeability/resistance measurements were performed (from day 0 to 17 after seeding), exceeding medium was discarded to restore quasi-ALI conditions, otherwise, after particle treatment, TEER measurement was followed by Resazurin assay.

#### Resazurin assay on transwells-grown A549 cells

Briefly, after measuring epithelial resistance (TEER), mitochondrial/metabolic activity was measured via Resazurin assay by adding 50 µl of resazurin 0.15 mg/ml (Roth) in PBS (Sigma) in the apical compartment of each well and incubating the cells for 2 h. Then 100 µl of supernatants from the transwells apical compartment were transferred in duplicates to a 96-well plate and fluorescence was recorded using a Tecan Infinite F Nano + microplate reader (Ex: 560; Em: 590) with the gain set on optimal. Triton X-100 treated samples (0.1% v/v, Roche) served as control for minimum viability and untreated cells as 100% viability.

#### Surface tension measurement on transwells-grown A549 cells

The surface tension in quasi-ALI and submerged samples was determined using the drop spreading method [80, 81], on cells grown on transwells in the 12-well plates format. Surface tension was measured both at 17 days after seeding and 48 h after treatment. Briefly, before performing the test, the supernatant of submerged cells was discarded, and cells were let air-exposed for 1 h at humified conditions. This equilibration period was important to exclude that medium leftover could affect drop spreading and consequently surface tension estimation. Then, small droplets of 5 µl composed by a mixture of dimethylphtalate (Sigma) and normal octanol (Honeywell) (4:1, v/v ratio, stained with 4 mg/ml of crystal violet (Sigma)) were dropped onto the epithelia surface. The surface tension of the hypophase was then calculated by the ratio of the diameter (d) of the deposited droplet (measured 30 s post deposition) and the diameter (d0) of the droplet prior deposition, while it was still hanging from the pipette: d/d0. The ST was quantified from a calibration curve obtained for thin liquid substrates, which gives the relationship between d/d0 and the ST of the film. The droplet diameter was calculated with the program ImageJ using photographs taken with a digital camera mounted on a Leica MZ FLIII stereomicroscope. Since deposited drops were sometimes not perfectly circular, the used diameters were calculated from the average of 4 different measurements in four different positions.

#### Particle translocation assessment on transwells-grown A549 and Calu-3 cells

Particle translocation potential was determined on A549 (submerged and quasi-ALI) and Calu-3 cells (quasi-ALI) grown on 12-well plates transwells using a fluorometric measurement. In A549, 150 µl of medium from the apical and basolateral compartments of treated and untreated A549 cells were transferred in duplicates to a 96-well plate. Then fluorescence emission of the Europium was quantified using a Tecan Infinite F Nano+ (Ex: 340; Em: 613) with gain set on optimal. Untreated and treated cell-free transwells served as background control and control from maximum translocation respectively. In quasi-ALI Calu-3 culture, basal media was collected and the apical compartment was collected by rinsing with 200 µL HBSS^Ca2+/Mg2+^. A cell lysate was prepared by adding 100 µL of water for 15 min at -80 °C to the apical compartment. Lysates, medium from the apical and basolateral compartments of treated and untreated cells were stored at -20 °C before analysis. Then fluorescence was quantified in 96-well clear bottom microplate (µClear^®^, Greiner Bio-One, Dominique Dutscher, Bernolsheim France) using a FlexStation 3 multi-mode microplate reader (Ex: 264 nm; Em: 613 nm for the apical and basal media and Em: 614 nm for the cell lysate). A standard curve with PS-Eu was done in each corresponding media.

#### Surfactant protein (SP) quantification on transwells-grown A549 cells

Surfactant proteins quantification was performed both on the pellets and the supernatant of treated and untreated cells. Prior SP quantification via ELISA, proteins were isolated from the cell pellets. Briefly, treated and untreated A549 cells were washed once with PBS. The cells were then lysed with 300 µl Triton buffer containing 0.2% protease inhibitors and 0.2% phosphatase inhibitors and incubated on ice for 30 min. After centrifugation for 5 min at 13,000 rpm (4 °C), the supernatant was transferred to a new tube. Then protein concentration was determined using the Bradford assay. Then pellet samples, prior ELISA, were diluted to a comparable total protein concentration. A549 cell pellet and their supernatants were analysed using a quantitative sandwich ELISA. Assays were performed using SP-ELISA kits from MyBiosource (MBS4502605-96, SP-A; MBS2703500-96, SP-B; MBS4502613-96, SP-C; MBS4502615-96, SP-D; MBS1606900-96, SP-H; San Diego, CA, USA) and Cloud-Clone (SED755Hu, SP-G; Cloud-Clone Corp., Wuhan, China) according to manufacturer instructions. Absorbance was collected at 405 nm and 450 nm.

#### Preparation of transwells-grown A549 and Calu-3 cells for transmission electron microscopy (TEM)

After 48 h of treatment with PS-Eu particles, A549 cells grown on 12-well plate transwells were washed using PBS (Sigma) and fixed in a mixture of 4% paraformaldehyde and 2% glutaraldehyde in 0.1 M cacodylate buffer (pH 7.3) for 3 h at 4˚C. After an overnight rinsing in 0.33 M sucrose in 0.1 M cacodylate buffer at 4 ˚C, post-fixation was performed with 1% osmium tetroxide (OsO_4_) (Roth, Germany) and 0.8% potassium ferrocyanide (K_4_[Fe(CN)_6_] x 3 H_2_O) (Sigma-Aldrich, Germany) in 0.2 M cacodylate buffer for 30 min in the dark at room temperature. After post-fixation, cells were rinsed with 0.1 M cacodylate buffer (pH 7.3), dehydrated in an ethanol series (5 min in each ethanol concentration), first impregnated with a mixture (1:1) of ethanol and Epon resin (Serva Electrophoresis, Germany) and then with pure Epon resin. Polymerization was carried out for five days at temperatures ranging from 35 ˚C to 80 ˚C.

Semi-thin Sect. (1 μm thick) were stained with 1% toluidine blue and 2% borate in distilled water and viewed with a Nikon Eclipse TE bright-field microscope (Amsterdam, The Netherlands).

Ultrathin Sects. (50–60 nm thick) were collected on copper grids, contrasted with aqueous uranyl acetate for 20 min and lead citrate for 2 min, and examined at 80 kV with a Philips CM100 transmission electron microscope (Eindhoven, The Netherlands). An AMT CCD camera (USA) and AMT Digital Micrograph software were used to obtain digital images.

In Calu-3 instead, all steps were performed at room temperature, except when otherwise indicated. Cells were fixed sequentially in 0.1 M sodium cacodylate, pH 7.3 containing 1.5% paraformaldehyde and 2.5% glutaraldehyde for 1 h, then in the same buffer containing 1% OsO_4_ for 1 h (at 4 °C) and finally in 2% aqueous uranyl acetate for 1 h. Cells were dehydrated in an ascending series of ethanol (30%, 50%, 70%, 95%, and 3 × 100%, for 10 min each), then incubated sequentially in 1/3, 1/1 and 3/1 (v/v) ethanol/Epon mixtures (1 h each) before final embedding in pure Epon.

Ultrathin Sect. (80 nm) were imaged using a Jeol 1400 TEM (Jeol, Croissy-sur-Seine, France), operated at 120 keV and equipped with a RIO CMOS camera (Ametek SAS, Elancourt, France).

#### *Alamar Blue*™ *assay on transwells-grown Calu-3 cells*

AlamarBlue™ (AB, Thermo Fisher Scientific) was diluted (1:10 ratio) in HBSS^Ca2+/Mg2+^ (Gibco, Thermo Fisher Scientific) and 1 ml was added to the basal compartment of control and treated cultures. After 2 h incubation, 100 µl of basal medium were transferred to a 96-well clear bottom microplate (µClear^®^). Fluorescence measurements were carried out on a FlexStation 3 multi-mode microplate reader (Molecular Devices) with Ex/Em wavelengths of 545 nm and 590 nm. To account for differences in cell number we compared the mitochondrial transformation of Alamar Blue of each transwell to the activity before treatment.

#### Enzyme-linked lectin assay (ELLA) on transwells-grown Calu-3 cells

Glycoproteins were quantified in the apical secretome by the ELLA according to Sanchez-Guzman and co-workers [78]. 96-well high binding plates were coated with lectin from Triticum vulgaris (Sigma-Aldrich) and washed with 0.5 M NaCl and 0.1% Teen 20 in PBS 1x. A standard curve was prepared with Porcine stomach mucin (Sigma-Aldrich) diluted in HBSS^Ca2+/Mg2+^. Standards and samples were incubated for 1 h at 37 °C. Lectin detection was performed with Glycine max peroxidase conjugate (Sigma-Aldrich) by 1 h incubation at 37 °C. Tetramethylbenzidine (TMB) substrate reagent (R&D systems, Bio-Techne) was added for 1 h at room temperature and the enzymatic reaction was stopped with the 2 N Sulfuric Acid Stop Solution.

The absorbance was measured at 490 nm on a UV-vis plate reader (EL808 Biotek microplate reader).

#### Gene expression analysis - RT-qPCR on transwells-grown Calu-3 cells

Cell lysis and RNA extractions from control and treated cultures were done using the Nucleospin RNA/Protein Mini kit (Macherey-Nagel, Hoerdt, France) according to manufacturer’s instructions. Cells were lysed by adding 350 µl of the lysis buffer supplemented with 0.5 M dithiothreitol (Merck) and gently scraping with the pipette tip to detach and collect cells from the transwells membrane. Reverse transcription of the mRNAs was performed using multiscribe High-cDNA Capacity RT kits (Applied Biosystems, ThermoFisher Scientific) according to the manufacturer’s protocols. qPCR was done using LightCycler SYBR Green master mix (Roche Diagnostics, Meylan, France) with primers from Eurofins Genomics (Ebersberg, Germany). Reactions were carried out in sealed 384-well plates (Roche Diagnostics) in a LightCycler 480 thermocycler (Roche Diagnostics).

Gene expression was quantified using 2-∆∆Ct method to yield Log2 fold change of a gene relative to HPRT and UBC as housekeeping genes and normalized to the control at each timepoint. Table 2 shows the primers used.

**Table 2:** – RT-qPCR primers

Target	Forward	Reverse
MUC5AC	ACAGCGGTGACTTCGACACA	CCGATGCCTGCTCCCTGTTA
MUC5B	CCAACGTCACCTGCGTGAAC	AGGTGCCGTCAAAGGTGGAA
HPRT	GGCGTCGTGATTAGTGATG	CAGAGGGCTACAATGTGATG
UBC	CACTTGGTCCTGCGCTTGA	TTTTTTGGGAATGCAACAACTTT
SOD2	CGGTAGCACCAGCACTAGCA	TGATGTGAGGTTCCAGGGCG
IL-8	CTCTCTTGGCAGCCTTCCT	AATTTCTGTGTTGGCGCAGT
IL-6	ACAGCCACTCACCTCTTCAG	TGGAAGCATCCATCTTTTTC
HO-1	CAGGCAGAGAATGCTGAGTTC	GCTCTTCTGGGAAGTAGA
NQO-1	AAGAAAGGATGGGAGGTGGTCAGG	GCTTCTTTTGTTCAGCCACA
CCL5	CAGTCGTCTTTGTCACCCGAA	TCCCAAGCTAGGACAAGAGCA
MCP-1	TCAGCCAGATGCAATCAATG	TCCTGAACCCACTTCTGCTT
TNF-α	GCTCCCCAAGAAGACAGG	GCCAGAGGGCTGATTAGAG
TGF-β	GGATCTCTCTCCGACCTGCC	GAAATAACCTAGATGGGCGCG
ZO-1	TGCAGCCAAGGAAGGCTTAGA	GGTCAAGCAGGAAAAGGACGG

Table showing the primers used to measure the gene expression via RT-qPCR of different proteins in response to treatment with increasing concentrations of PS-Eu. The targets full name are: interleukin 8 (IL-8); interleukin 6 (IL-6); monocyte chemoattractant protein-1 (MCP-1); chemokine (C-C motif) ligand 5 (CCL5); tumor necrosis factor-α (TNF-α); transforming growth factor-β (TGF-β:); superoxide dismutase 2 (SOD2); heme-oxygenase 1 (HO-1); NAD(P)H quinone dehydrogenase 1 (NQO-1); hypoxanthine guanine phosphoribosyltransferase (HPRT); ubiquitin C (UBC); zonula occludens 1 (ZO-1); mucin 5 A (MUC5A); mucin 5B (MUC5B)

#### Raman microscopy on transwells-grown Calu-3 cells

Calu-3 cells exposed at quasi-ALI to PS-Eu nanoplastics for 24 h at 45 µg/cm² were fixed with PFA 4% for 20 min at room temperature and rinsed with PBS before imaging. The transwell membrane was placed on fused silica substrate (ESCO Optics, UK) with cells facing the objective lens. Raman images were acquired on a WITec alpha300 RA inverted Raman microscope (Oxford Instruments) with a Zeiss 100x oil immersion objective (NA 1.3) using an excitation wavelength of 532 nm and a grating of 600 g/mm. Exposure time was set to 0.1 s and laser power to 10 mW. An area of 30 × 30 μm was analysed. 3D Raman images were taken using a depth step of 1 μm. Raman images were analysed using WITec Project 5 Plus software for cosmic ray removal, baseline subtraction, and spectral analysis.

### Statistical analysis

Statistical tests were performed using the GraphPad Prism software version 10.0.2 (La Jolla, CA, USA). The normality of the data was tested with the Shapiro-Wilk test. For comparing different groups, one-way ANOVA was performed with Kruskal-Wallis test or Wilcoxon–Mann–Whitney test with Dunn’s multiple comparison for not normally distributed samples, or one-way ANOVA with Dunnett’s and Sidak tests for normally distributed samples. To compare different groups at different timepoints or different culture systems (quasi-ALI vs. submerged), two-way ANOVA was used with Dunnett’s multiple comparison test. Results were considered significant if *p* < 0.05 (*, #, §), *p* < 0.01(**, ##, §§), *p* < 0.001(***, ###, §§§), or *p* < 0.0001(****, ####, §§§§).

## Electronic supplementary material

Below is the link to the electronic supplementary material.

Supplementary Material 1: DLS graph showing the impact of FBS-free medium on particle aggregation and sedimentation.

Supplementary Material 2: Scheme summarizing the different culture systems and assays used to investigate PS-Eu particle impact on 2D- and 3D-grown A549 cells and TEM images showing transwells sections.

Supplementary Material 3: 3D Raman scan of quasi-ALI-grown Calu-3 cells exposed to PS-Eu for 24 h, plus IL-8 and IL-6 ELISA protocol and results. The cross-section in the XY and Z planes of the 3D Raman image with cells (green) and PS-Eu (red) are shown. The transwell membrane was placed with the apical side towards the bottom and the basolateral side of the transwell membrane towards the top. Scale bar 6 μm.

Supplementary Material 4: Scheme summarizing the different culture systems and assays used to investigate PS-Eu particle impact on 2D- and 3D-grown cultured human airway epithelial cells (Calu-3).

## Data Availability

The datasets used and/or analysed during the current study are available from the corresponding author on reasonable request.
