# Supplementary material for: Pulmonary hazards of nanoplastic particles: a study using polystyrene in in vitro models of the alveolar and bronchial epithelium
Source: J Nanobiotechnology. 2025 May 28;23:388. doi: 10.1186/s12951-025-03419-6 (PMC12117733; doi:10.1186/s12951-025-03419-6)
Supplement: Supplementary file 2 — Supplementary Material 2: Scheme summarizing the different culture systems and assays used to investigate PS-Eu particle impact on 2D- and 3D-grown A549 cells and TEM images showing transwells sections. [file 12951_2025_3419_MOESM2_ESM.pdf]

Pulmonary Hazards of Nanoplastic Particles: A Study Using Polystyrene in *in Vitro* Models of the Alveolar and Bronchial Epithelium

Sara Michelini [a], Safaa Mawas [b], Ema Kurešepi [a], Francesco Barbero [c], Katarina Šimunović [d], Dorian Miremont [b], Stéphanie Devineau [b], Martin Schicht [e], Victor Ganin [f], Charlotte Izabelle [h], Øyvind P Haugen [g], Anani Komlavi Afanou [g], Shan Zienolddiny-Narui [g], Katharina Jüngert [e], Neža Repar [a], Ivana Fenoglio [c], Barbara Šetina Batić [f], Friedrich Paulsen [e], Ines Mandić-Mulec [d], Sonja Boland [b], Andreja Erman [i], Damjana Drobne [a+]

[a] *University of Ljubljana, Biotechnical faculty, Department of Biology, Jamnikarjeva ulica 101, 1000 Ljubljana, Slovenia*
[b] *Université Paris Cité, CNRS, Unité de Biologie Fonctionnelle et Adaptative, F-75013 Paris, France.*
[c] *University of Torino, Department of Chemistry, Laboratory of Toxicity and Biocompatibility of Materials, Torino, Italy*
[d] *University of Ljubljana, Biotechnical faculty, Department of Microbiology, Jamnikarjeva ulica 101, 1000 Ljubljana, Slovenia*
[e] *Friedrich-Alexander-University of Erlangen-Nürnberg, Institute of Functional and Clinical Anatomy, Erlangen, Germany*
[f] *Institute of Metals and Technology, Lepi pot 11, 1000 Ljubljana, Slovenia*
[g] *STAMI, National Institute of Occupational Health, Gydas Vei 8, 0363 Oslo, Norway*
[h] *Université Paris Cité, CNRS UAR612, Inserm US25, Cellular and Molecular Imaging facility, F-75006, Paris, France.*
[i] *University of Ljubljana, Faculty of Medicine, Institute of Cell Biology, Vrazov trg 2, 1000 Ljubljana, Slovenia*

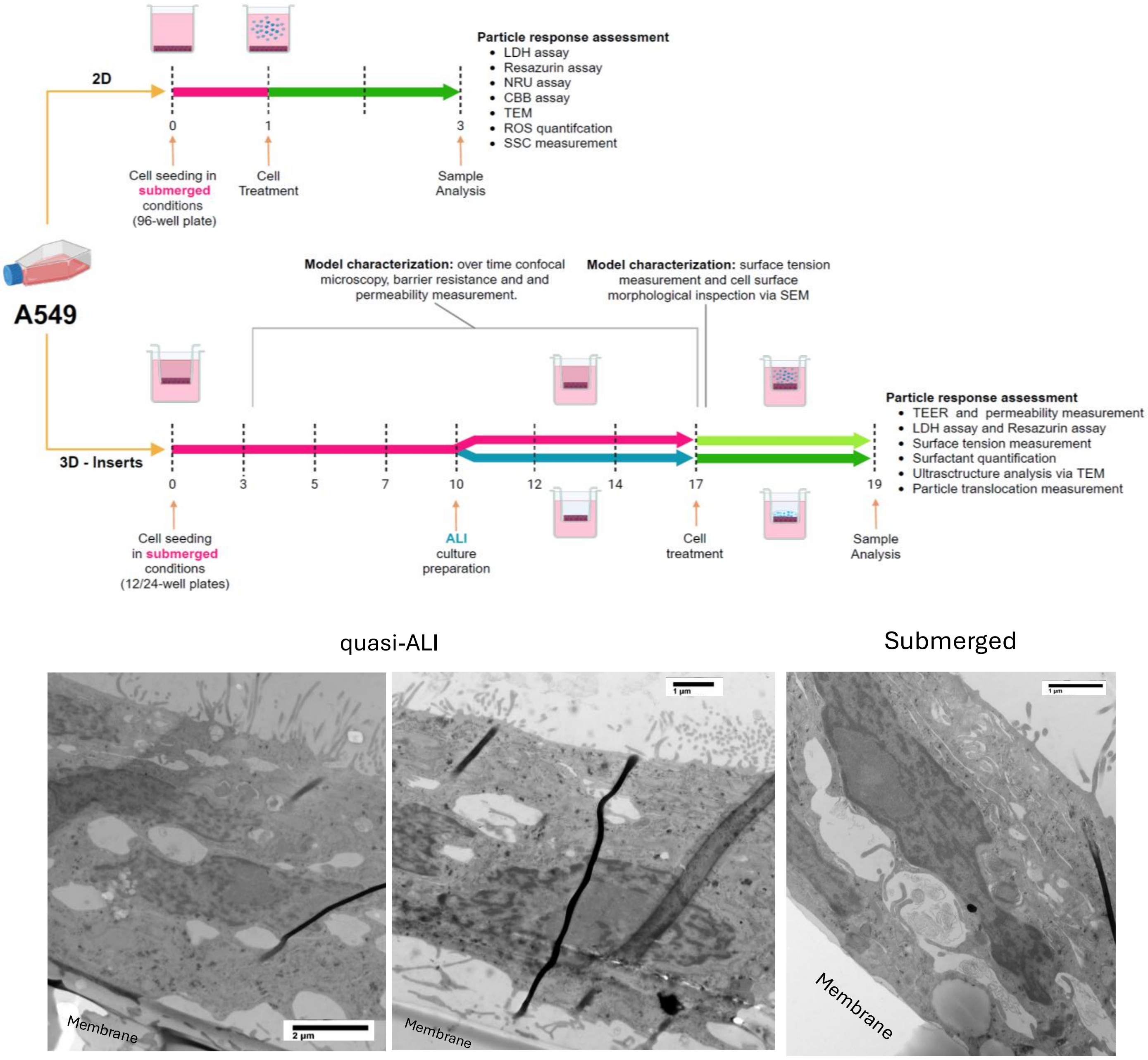

***Additional File 2: Top)** scheme summarizing the different culture systems and assays used to investigate PS-Eu particle impact on 2D and 3D grown A549 cells. **Bottom):** TEM images of A549 cells grown in ALI or Submerged conditions. **Abbreviations**: TEER, transepithelial electrical resistance; ALI: quasi air-liquid interface; LDH: Lactate dehydrogenase; TEM and SEM: Transmission and scanning electron microscopy; NRU: neutral red uptake; ROS: reactive oxygen species; SSC: side-scatter; CBB: Coomassie Brilliant Blue; A549: adenocarcinoma human alveolar basal epithelial cells.*
