# Supplementary material for: Pulmonary hazards of nanoplastic particles: a study using polystyrene in in vitro models of the alveolar and bronchial epithelium
Source: J Nanobiotechnology. 2025 May 28;23:388. doi: 10.1186/s12951-025-03419-6 (PMC12117733; doi:10.1186/s12951-025-03419-6)
Supplement: Supplementary file 3 — Supplementary Material 3: 3D Raman scan of quasi-ALI-grown Calu-3 cells exposed to PS-Eu for 24 h, plus IL-8 and IL-6 ELISA protocol and results. The cross-section in the XY and Z planes of the 3D Raman image with cells (green) and PS-Eu (red) are shown. The transwell membrane was placed with the apical side towards the bottom and the basolateral side of the transwell membrane towards the top. Scale bar 6 μm. [file 12951_2025_3419_MOESM3_ESM.pdf]

Pulmonary Hazards of Nanoplastic Particles: A Study Using Polystyrene in *in Vitro* Models of the Alveolar and Bronchial Epithelium

Sara Michelini [a], Safaa Mawas [b], Ema Kurešepi [a], Francesco Barbero [c], Katarina Šimunović [d], Dorian Miremont [b], Stéphanie Devineau [b], Martin Schicht [e], Victor Ganin [f], Charlotte Izabelle [h], Øyvind P Haugen [g], Anani Komlavi Afanou [g], Shan Zienolddiny-Narui [g], Katharina Jüngert [e], Neža Repar [a], Ivana Fenoglio [c], Barbara Šetina Batić [f], Friedrich Paulsen [e], Ines Mandić-Mulec [d], Sonja Boland [b], Andreja Erman [i], Damjana Drobne [a+]

[a] *University of Ljubljana, Biotechnical faculty, Department of Biology, Jamnikarjeva ulica 101, 1000 Ljubljana, Slovenia*
[b] *Université Paris Cité, CNRS, Unité de Biologie Fonctionnelle et Adaptative, F-75013 Paris, France.*
[c] *University of Torino, Department of Chemistry, Laboratory of Toxicity and Biocompatibility of Materials, Torino, Italy*
[d] *University of Ljubljana, Biotechnical faculty, Department of Microbiology, Jamnikarjeva ulica 101, 1000 Ljubljana, Slovenia*
[e] *Friedrich-Alexander-University of Erlangen-Nürnberg, Institute of Functional and Clinical Anatomy, Erlangen, Germany*
[f] *Institute of Metals and Technology, Lepi pot 11, 1000 Ljubljana, Slovenia*
[g] *STAMI, National Institute of Occupational Health, Gydas Vei 8, 0363 Oslo, Norway*
[h] *Université Paris Cité, CNRS UAR612, Inserm US25, Cellular and Molecular Imaging facility, F-75006, Paris, France.*
[i] *University of Ljubljana, Faculty of Medicine, Institute of Cell Biology, Vrazov trg 2, 1000 Ljubljana, Slovenia*

Additional file 3:

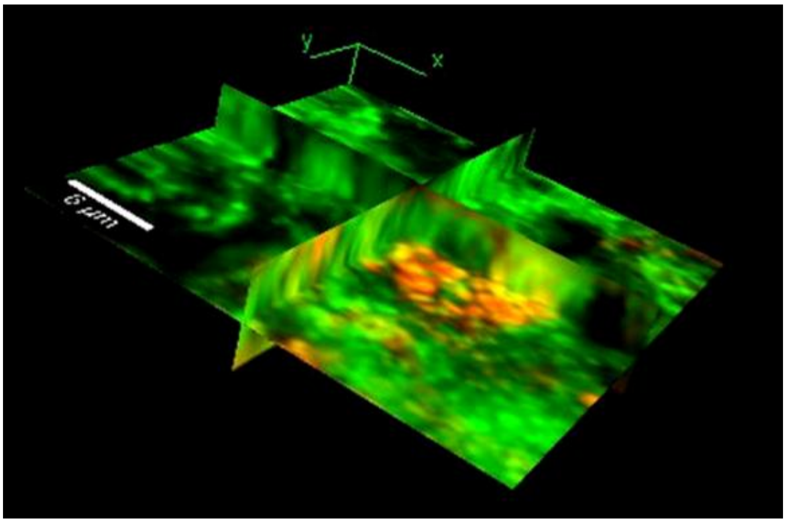

Figure 1*: 3D Raman scan of ALI-grown Calu-3 cells exposed to PS-Eu for 24h. The cross-section in the XY and Z planes of the 3D Raman image with cells (green) and PS-Eu (red) are shown. The insert was placed with the apical side towards the bottom and the insert towards the top. Scale bar 6 µm.*

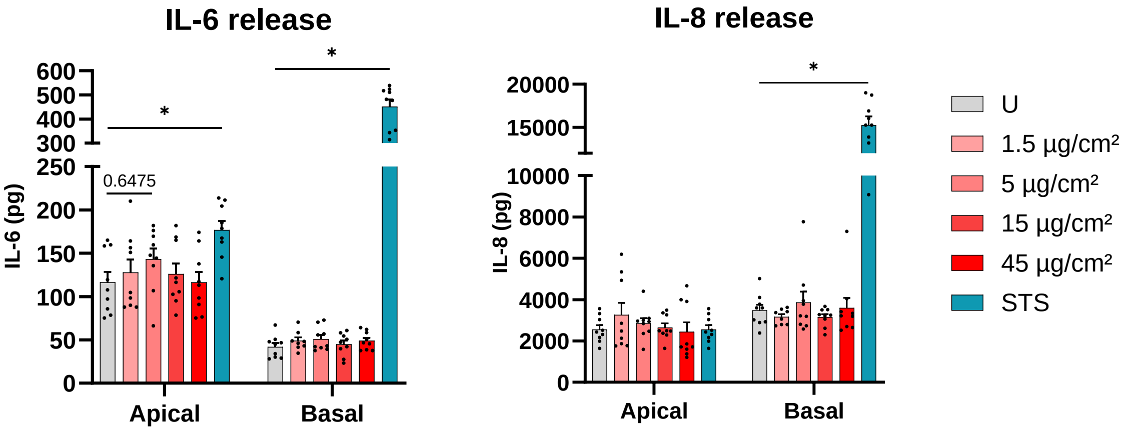

Figure 2*: Quantification of IL-6 and IL-8 pro-inflammatory cytokines release in the apical and basolateral secretomes of quasi-ALI-grown Calu-3 cells. Calu-3 cells were cultured in ALI conditions for 7 days before 24 h treatment with droplets (18 µL/cm^2) of PS-Eu suspension. Particles were applied from 1.5 to 45 µg/cm2 and 0.75 µM staurosporine (STS) was used as positive control. After exposure, the apical secretome was rinsed twice with 0.2 mL of HBSSCa2+/Mg2+ and collected and the basolateral secretome was collected for cytokines quantification by ELISA. 3 independent experiments with 3 technical replicates were performed and reported as mean ± standard error of mean. Statistical analysis was performed using a one-way ANOVA with the Kruskal-Wallis test and Dunn's multiple comparisons. Abbreviations: ELISA: enzyme-linked immunosorbent assay, IL-6: Interleukin 6, IL-8: Interleukin 8.*

Protocols:

ELISA

The apical secretome was rinsed with 200 µL of HBSS$^{Ca2+/Mg2+}$and the basolateral secretome was collected after exposure and both secretomes were stored at -20°C, to quantify released apical and basolateral pro-inflammatory cytokines IL-6, IL-8 using DuoSet ELISA kits (DY206, DY08, R&D Systems Biotechne, Noyal Chantillon sur Seiche, France), following the manufacturer’s instructions. The optical density was measured at 450 and 595 nm using the BioTeck EL808 spectrophotometer.
